# Catalytic mechanism and pH dependence of a methyltransferase ribozyme (MTR1) from computational enzymology

**DOI:** 10.1093/nar/gkad260

**Published:** 2023-04-18

**Authors:** Erika McCarthy, Şölen Ekesan, Timothy J Giese, Timothy J Wilson, Jie Deng, Lin Huang, David M J Lilley, Darrin M York

**Affiliations:** Laboratory for Biomolecular Simulation Research, Institute for Quantitative Biomedicine and Department of Chemistry and Chemical Biology, Rutgers University, Piscataway, NJ 08854, USA; Laboratory for Biomolecular Simulation Research, Institute for Quantitative Biomedicine and Department of Chemistry and Chemical Biology, Rutgers University, Piscataway, NJ 08854, USA; Laboratory for Biomolecular Simulation Research, Institute for Quantitative Biomedicine and Department of Chemistry and Chemical Biology, Rutgers University, Piscataway, NJ 08854, USA; Nucleic Acid Structure Research Group, MSI/WTB Complex, The University of Dundee, Dundee, Dow Street, Dundee DD1 5EH, UK; Guangdong Provincial Key Laboratory of Malignant Tumor Epigenetics and Gene Regulation, Guangdong–Hong Kong Joint Laboratory for RNA Medicine, Sun Yat-sen Memorial Hospital, Sun Yat-sen University, Guangzhou 510120, China; Medical Research Center, Sun Yat-sen Memorial Hospital, Sun Yat-sen University, Guangzhou 510120, China; Guangdong Provincial Key Laboratory of Malignant Tumor Epigenetics and Gene Regulation, Guangdong–Hong Kong Joint Laboratory for RNA Medicine, Sun Yat-sen Memorial Hospital, Sun Yat-sen University, Guangzhou 510120, China; Medical Research Center, Sun Yat-sen Memorial Hospital, Sun Yat-sen University, Guangzhou 510120, China; Nucleic Acid Structure Research Group, MSI/WTB Complex, The University of Dundee, Dundee, Dow Street, Dundee DD1 5EH, UK; Laboratory for Biomolecular Simulation Research, Institute for Quantitative Biomedicine and Department of Chemistry and Chemical Biology, Rutgers University, Piscataway, NJ 08854, USA

## Abstract

A methyltransferase ribozyme (MTR1) was selected *in vitro* to catalyze alkyl transfer from exogenous *O*^6^-methylguanine (*O*^6^mG) to a target adenine N1, and recently, high-resolution crystal structures have become available. We use a combination of classical molecular dynamics, *ab initio* quantum mechanical/molecular mechanical (QM/MM) and alchemical free energy (AFE) simulations to elucidate the atomic-level solution mechanism of MTR1. Simulations identify an active reactant state involving protonation of C10 that hydrogen bonds with *O*^6^mG:N1. The deduced mechanism involves a stepwise mechanism with two transition states corresponding to proton transfer from C10:N3 to *O*^6^mG:N1 and rate-controlling methyl transfer (19.4  kcal·mol^−1^ barrier). AFE simulations predict the p*K*_a_ for C10 to be 6.3, close to the experimental apparent p*K*_a_ of 6.2, further implicating it as a critical general acid. The intrinsic rate derived from QM/MM simulations, together with p*K*_a_ calculations, enables us to predict an activity–pH profile that agrees well with experiment. The insights gained provide further support for a putative RNA world and establish new design principles for RNA-based biochemical tools.

## Graphical Abstract

**Graphical Abstract:**
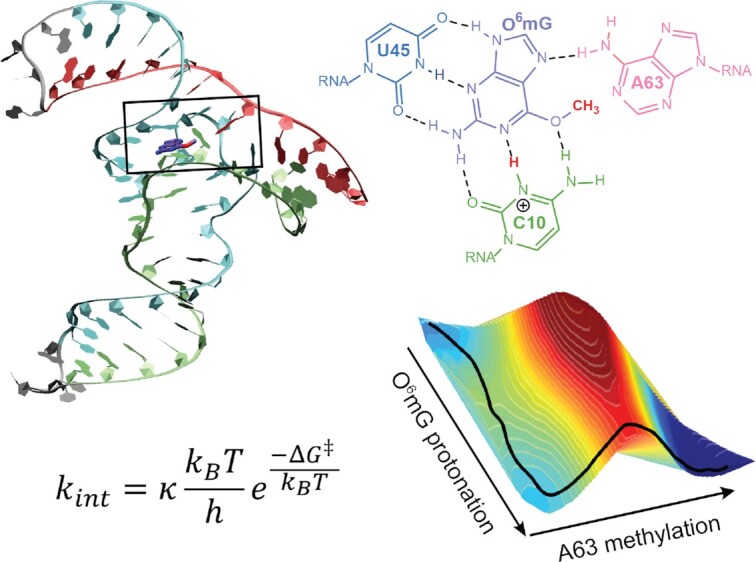
Mechanistic study of a novel artificially engineered methyltransferase ribozyme (MTR1), catalyzing site-specific methylation of adenine, utilizing state-of-the-art computational tools.

## INTRODUCTION

Small self-cleaving ribozymes use unique strategies to maximize the reactivity of their limited chemical library of four nucleobases in order to catalyze phosphoryl transfer reactions (1–5). In the context of the RNA world hypothesis (6,7), these reactions are potentially evolutionary vestiges of chemical functions that have since been overtaken by proteins, begging the question of whether ancient RNAs once had the ability to catalyze more diverse reactions as precursors to modern protein counterparts (8–10), and how their mechanistic strategies may be reflected in modern biology. Evaluating this catalytic range is critical to rationalize evolutionary theories and may also shed light onto relationships between ribozyme classes (3,11), and between ribozymes and riboswitches (12–15).

Of particular interest from an evolutionary perspective is the ability of RNA molecules to catalyze C–C and C–N bond formation that would be essential for nucleic acid synthesis and early metabolic transformations. *In vitro* selection methods have resulted in the generation of ribozymes that catalyze diverse reactions with potential applications in gene therapy and as biosensors (16,17), including Diels–Alder reaction (18,19), aldol condensation (20), alkylation (16) and Michael addition (21); however, the lack of available structural information has made these reactions difficult to interpret and exploit. Enabled by these experimental data, computational work has explored the mechanism (22) and magnesium-dependent active site conformational selection (23) in the Diels–Alderase ribozyme. Pioneering work by Wilson and Szostak (16) identified the first self-alkylating ribozyme that catalyzed N–C bond formation in a self-biotinylation reaction, which has only recently been structurally characterized (24,25). In addition, guanine N7 methylation has been exhibited by an artificially engineered SAM-dependent methyltransferase ribozyme (SMRZ-1) (26), and cytosine N3 methylation has been demonstrated to some degree in the prequeuosine^1^ (preQ_1_) riboswitch (27).

Recently, a methyltransferase ribozyme (MTR1) has evolved *in vitro* (28) that binds *O*^6^-methylguanine (*O*^6^mG) and catalyzes the methylation of a target adenine (A63) at the N1 position. *N*^1^-Methyladenosine modification has structural and functional roles in tRNA (29) and rRNA (30) as it modifies the Watson–Crick face and carries a positive charge under physiological conditions (31). Crystal structures for MTR1 have been determined (32,33) that, together with biochemical experiments, have provided insights into catalytic mechanism. These studies open the door for rigorous theoretical work aimed at providing an atomic-level interpretation of the current body of experimental data, and ultimately gaining deep insight into mechanism and the factors that regulate activity. Of key importance is the elucidation of the rate-controlling transition state ensemble that, while not directly accessible by experiments, can be investigated computationally (34). Such a predictive-level understanding is instrumental for the design of new ribozyme-based biotechnology and therapeutics, and may further shed light onto inherent differences between engineered ribozymes that have evolved rapidly *in vitro* under artificial selection pressures and biological ribozymes that have evolved gradually *in vivo* through natural selection.

Herein, we employ a computational enzymology approach (35,36) to study the catalytic mechanism of the MTR1 ribozyme. To our knowledge, this is the first in-depth computational mechanistic study of an artificially engineered alkyl transfer RNA enzyme. Molecular dynamics (MD) simulations are used to characterize the structure and dynamics of the ribozyme in solution and identify the dynamical active state ensemble (i.e. the conformation and protonation state capable of catalyzing the chemical reaction). Departing from the active state, combined quantum mechanical/molecular mechanical (QM/MM) simulations are used to explore the chemical mechanism and identify the free energy pathway and transition state ensembles. Alchemical free energy (AFE) simulations are used to examine the electrostatic environment in the active site and predict p*K*_a_ shifts of key nucleotides that plausibly give rise to the experimentally observed activity–pH profiles. Our results suggest a remarkably sophisticated mechanism involving general acid catalysis by an active site cytosine nucleotide (C10), as was suggested by experimental work (32,33), facilitated by electrostatic interactions (5) and dynamical motions (37,38) in the active site. Taken together, the results of this work may provide new insights that facilitate nucleic acid enzyme design efforts and help further our fundamental understanding of the catalytic chemical landscape of ribozymes.

## MATERIALS AND METHODS

Here, we provide a summary of the main calculations performed in the present work, the full technical details for which are described in the ‘Detailed Computational Methods’ section of the Supplementary Data. Departing from the crystal structure of MTR1 with a bound guanine ligand and methylated target adenine [PDB ID 7V9E (32)], we construct a solution-phase model of the wild-type (WT) ribozyme in its reactant (*O*^6^mG-bound) state and performed MM simulations to predict the dynamical ensemble in solution. These MM simulations also provide a starting point from which to identify a plausible ‘active state’ in solution capable of catalyzing the chemical reaction. The active state is usually a rare state that must satisfy certain geometrical and protonation state requirements (37). In the present case, this involves alignment of the nucleophile (A63:N1), electrophilic carbon (*O*^6^mG:Cm) and leaving group (*O*^6^mG:O6), as well as appropriate protonation state of the implicated general acid (C10:N3).

The system was prepared by solvating the MTR1 [PDB ID 7V9E (32)] crystal structure coordinates in a truncated octahedron (90.2 Å real-space lattice vector lengths) with 18,250 TIP4P-Ew water molecules (39), 133 Na^+^ ions and 47 Cl^−^ ions to neutralize the ribozyme charge and achieve a physiological ion concentration of 140 mM NaCl. We used the monovalent ion parameters designed for use with the TIP4P-Ew water model (40). Electrostatics were performed with the particle mesh Ewald method (41,42). We performed five independent 150 ns MD simulations of the full MTR1 (32) using the ff99OL3 (43,44) RNA force field. Solute coordinate heavy-atom root-mean-square deviation (RMSD) with respect to the starting structure was analyzed to determine a single cutoff time that encapsulated the structurally unequilibrated part of the trajectories (50 ns), after which the RMSD was observed to fluctuate stably and analysis could be made over the last 100 ns for each independent simulation (see Supplementary Table S1 and Supplementary Figure S1).

Next, departing from the identified active state ensemble in solution, we performed combined QM/MM simulations with long-ranged electrostatics treated rigorously with the linear-scaling ambient-potential composite Ewald method (45). QM/MM umbrella sampling simulations were used to explore the mechanism of the adenine N1 methylation reaction in the WT ribozyme, and analyzed using a generalized multidimensional variational free energy profile method (46). The reactant state is characterized by a protonated C10 at the N3 position and a methylated (Cm) ligand oxygen *O*^6^mG:O6. The product state is characterized by a protonated ligand nitrogen *O*^6^mG:N1 and a methylated A63 at the N1 position. The mechanism is explored with two reaction coordinates that represent the proton transfer (*ξ*_PT_) and methyl transfer (*ξ*_MT_):


(1) $$\begin{equation*} \xi _{\text{PT}} = \vert {R}_{\text{C10:N3}}-{R}_{\text{H}}\vert - \vert {R}_{\text{{\it O}\textsuperscript {6}mG:N1}}- {R}_{\text{H}}\vert ,\end{equation*}$$

(2) $$\begin{equation*} \xi _{\text{MT}} = \vert {R}_{\text{\text{{\it O}\textsuperscript {6}mG:O6}}}- {R}_{\text{Cm}}\vert - \vert {R}_{\text{A63:N1}}-{R}_{\text{Cm}}\vert .\end{equation*}$$

To get an overview of the two-dimensional (2D) free energy surfaces (FES) and enumerate plausible mechanistic pathways, we performed QM/MM umbrella sampling with the DFTB3 (47–49) semi-empirical Hamiltonian using the 3OB-3-1 parameter set (50). The simulations were chosen to form a regular grid of 390 simulations that spanned the ranges *ξ*_PT_ ∈ [−1.5, 1.5] and *ξ*_MT_ ∈ [−2.5, 2.5] in steps of 0.2 Å. The minimum free energy pathways of the WT and C10U mutant ribozymes were both found to correspond to a stepwise mechanism in which proton transfer precedes the methyl transfer. Alternative pathways in which the order of these steps was reversed or occurred in concert were also identified, but with considerably higher barriers. We thus performed much more computationally intensive PBE0/6-31G* QM/MM umbrella sampling of the reactions using the umbrella sampling finite temperature string method (51) to obtain a more accurate quantitative estimate of the minimum free energy path and rate-limiting transition state barrier.

Finally, we estimate the activity–pH profile of the WT ribozyme using a two-state, noncooperative model (36,52):


(3) $$\begin{eqnarray*} k_{\text{obs}}&=& k_{\text{int}} \cdot f_{\text{obs}}(\text{pH}) =k_{\text{int}}/(1+10^{\text{p}K_{{\rm a,B}}-\text{pH}}\nonumber\\ && +\,10^{\text{pH}-\text{p}K_{{\rm a,A}}}+10^{\text{p}K_{{\rm a,B}}-\text{p}K_{{\rm a,A}}}), \end{eqnarray*}$$

where *k*_obs_ is the observed rate constant, *k*_int_ is the intrinsic rate constant as shown in Equation (S1) and *f*_obs_(pH) is the pH-dependent fraction (probability) the ribozyme is in the active protonation state and written out as the inverse of the partition function (shown in parentheses as the denominator) enumerating the protonation states of the acid (A) and base (B) that affect the activity–pH profile. Specifically, p*K*_a,A_ and p*K*_a,B_ refer to the apparent p*K*_a_ of the acid and base, respectively. In the present context, the acid is presumed to be the C10 nucleotide. There is no general base catalyst in the reaction; nonetheless, a protonation event of a functionally-important base was identified from the experimental activity–pH profiles through the apparent p*K*_a_ values. In the present work, our calculations suggest a plausible candidate for this base apparent p*K*_a_ to be A63.

The value of p*K*_a,A_ (and analogously p*K*_a,B_) is estimated by correcting the experimental $\text{p}K_{{\rm a,A}}^{\text{expt.}}$ in solution with a Δp*K*_a,A_ shift caused by the ribozyme environment:


(4) $$\begin{equation*} \text{p}K_{{\rm a,A}}=\text{p}K^{\text{expt.}}_{{\rm a,A}}+\Delta \text{p}K_{{\rm a,A}} .\end{equation*}$$

The Δp*K*_a,A_ shift is calculated from AFE simulations (53,54), evaluated from the difference in free energies associated with deprotonating the C10 nucleotide in the ribozyme and aqueous environments:


(5) $$\begin{equation*} \Delta \text{p}K_{{\rm a,A}}=\frac{\Delta G_{\text{RNA}}({\rm AH}^{+}\rightarrow {\rm A})-\Delta G_{\text{aq}}({\rm AH}^{+}\rightarrow {\rm A})}{RT\ln (10)} .\end{equation*}$$

The p*K*_a_ values of cytosine and adenine in solution have been experimentally determined to be 4.2 (55) and 3.5 (56), respectively. The recently developed smoothstep softcore potentials (57) were used to create intermediate states that connect the protonated and deprotonated systems, and production statistics were gathered for 5 ns/state using an alchemical enhanced sampling (54,58) approach. Full details of the MM and AFE simulations and other computational methods are provided in the Supplementary Data.

## RESULTS AND DISCUSSION

### Structure and dynamics of MTR1 in solution

The first goal of the present work is to characterize the dynamical ensemble of the active state of MTR1 in solution, i.e. the substrate-bound state that is competent to carry out the catalytic chemical steps of the reaction. Our simulations depart from the 2.3 Å resolution MTR1 structure 7V9E (32) (the highest resolution structure currently available). The electron density map indicates the presence of a methyl group at the N1 position of A63, suggesting the ribozyme is active in the crystal, or during the crystallization process, and the resolved structure represents the product form. In constructing a model of the substrate-bound reactant state, we considered that in the crystal the N1 of guanine and N3 of C10 are separated by 2.7 Å, indicating a potential hydrogen bond interaction, and consistent with the supposition that a proton was possibly transferred from C10H^+^ to *O*^6^mG. We thus considered this as a plausible active state model and performed a series of five independent simulations, the last 100 ns of which were shown to be equilibrated and used for analysis (Supplementary Table S1 and Supplementary Figure S1), departing from the crystal structure but with C10:N3 protonated and the methyl group connected to *O*^6^mG as expected for the reactant state. As will be shown below, this protonation in the active state is supported by activation barriers and p*K*_a_ shifts predicted from QM/MM and AFE simulations, respectively.

**Figure 1.**
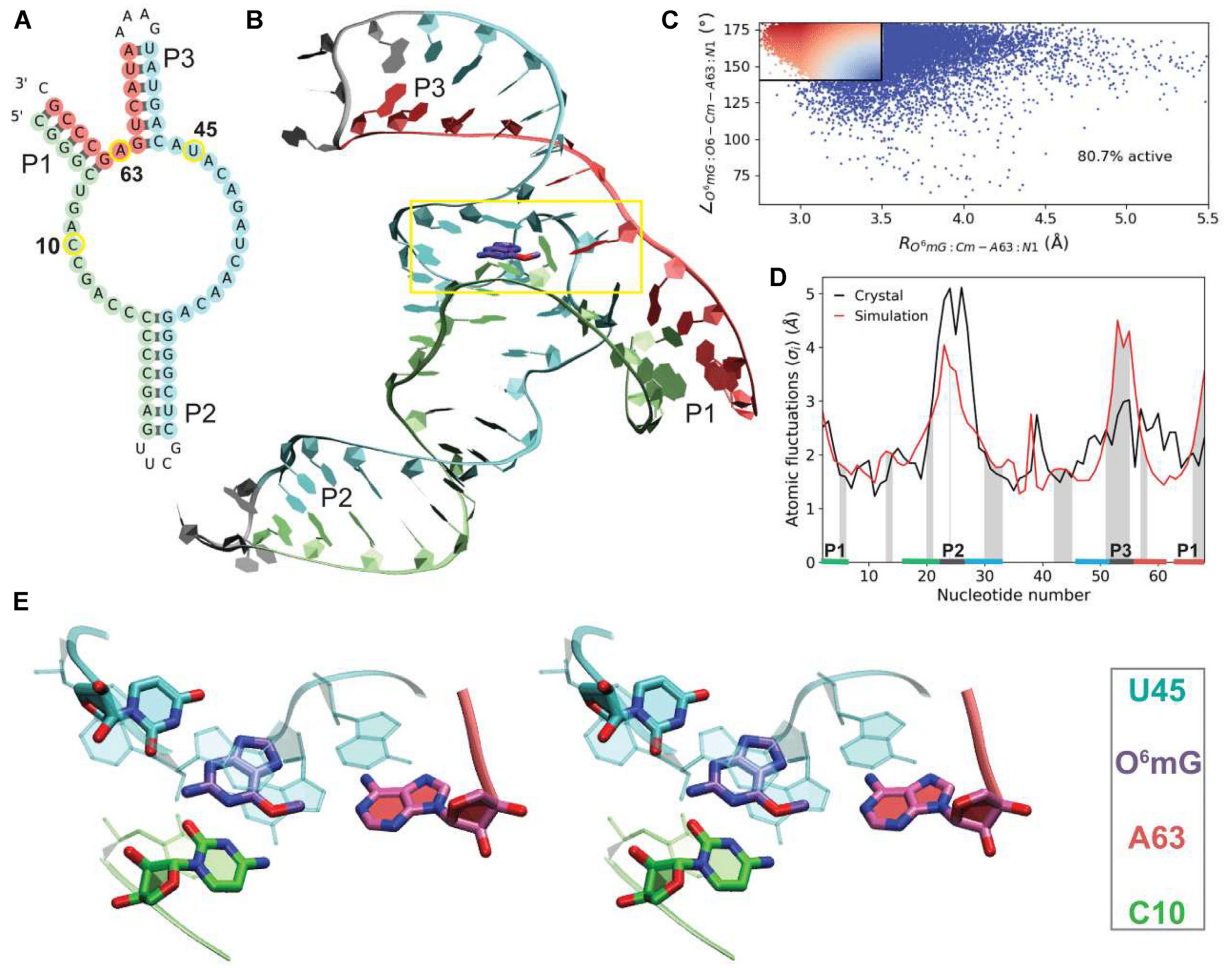
Active structure of MTR1 in solution. (**A**) Secondary structure of MTR1 colored by helical regions P1, P2 and P3 with loop regions in gray. (**B**) Average solution structure over the last 10 ns of simulation with *O*^6^mG (purple) bound. (**C**) Distribution of nucleophile–electrophile angle (*O*^6^mG:O6–Cm–A63:N1) and distance (*O*^6^mG:Cm–A63:N1) colored from least catalytically fit (blue) to most catalytically fit (red). Fitness scores were assigned such that the angle must be between 140° and 180° and distance between 3.50 and 2.75 Å (as denoted by the upper left box), otherwise a score of zero (blue) was given (see Supplementary Data for a detailed explanation of scoring). (**D**) Atomic fluctuations averaged per nucleotide, iterating over *i* nucleotides $( \langle \sigma_i \rangle )$, derived from analysis of 5 × 100 ns MD simulations (red) and compared with fluctuations estimated from the crystallographic *B*-values (black) with correlation coefficient of 0.62. Helical arms and loop regions are highlighted on the horizontal axis, and shaded regions indicate crystal contacts. (**E**) Stereo view of the active site nucleotides of MTR1 in solution with coloring corresponding to panels (A) and (B).

Key results from the MD simulations are summarized in Figure 1. The MTR1 structure [PBD ID 7V9E (32)] is a three-way junction comprising the three helical arms P1, P2 and P3, formed from a single RNA strand connected by addition of a GNRA tetraloop to the end of the P3 helix (Figure 1A and B). The active site is formed at the three-way junction and has a core comprised of three functionally-important nucleotides that bind the *O*^6^mG substrate through hydrogen bond interactions (Figure 1E): C10, U45 and A63. C10 and A63, as will be examined in more detail below, also play chemical roles in catalysis. C10 is stabilized by base stacking with A9 and C11, and A63 is stabilized by base stacking with A40. The *O*^6^mG binding pocket is sandwiched from above and below by planes formed by the A9·A46·A40 base triple and by the C11·G41 Watson–Crick base pair.

The average structures from the simulations are similar to the crystal structure (32) (not including the transferred methyl group), particularly for the functionally-important active site nucleotides with RMSD values ranging from 0.63 to 0.88 Å (Supplementary Table S1). Only one simulation (sim 1) has an overall RMSD >2.9 Å from the crystal structure and appears to be somewhat of an outlier. Figure 1D compares atomic fluctuations (59) ($\langle \sigma_i \rangle$, averaged for each nucleotide *i*) from the solution simulations and derived from the crystallographic data. The fluctuations are generally similar, with the largest discrepancies in the P1 (nucleotides 65–69) and P3 regions where stabilizing crystal contacts (indicated by shading) are not present in solution. The dynamical ensemble in solution has the largest fluctuations in the P1 (3′ and 5′ paired termini), P2 (loop) and P3 (loop) regions due to lack of base pairing and/or stacking. The ability of RNA force fields to model loop conformations [e.g. hairpins (60,61) and tetraloops (62–64)] is an ongoing challenge. Given the number of crystal contacts in the P2 and P3 loops in the experimental structure, it is hard to determine whether RNA force field issues might also be a contributing factor to the large fluctuations observed in these regions in solution. Overall, these analyses suggest that the active site structure is preserved, there are no significant conformational changes that occur in the relaxation from the crystalline environment to solution and only moderate deviations in the dynamical fluctuations are observed.

Of key importance for catalysis is the degree to which the substrate and active site nucleotides sample ‘active’ conformations relevant for the chemical steps of the reaction to occur. In the present case, this involves the nucleophilic attack of the A63:N1 to the *O*^6^mG:Cm, and departure of the *O*^6^mG:O6 leaving group. The requirements for this to occur effectively are that the *O*^6^mG:Cm–A63:N1 distance is sufficiently close, and that the A63:N1–*O*^6^mG:Cm–*O*^6^mG:O6 angle (*θ*_inl_) is ‘inline’ (sufficiently close to 180°). Previous theoretical work (36–38) has established that a useful heuristic measure of ‘inline fitness’ of the dynamical ensemble for similar nucleophilic attack reactions is to restrict the nucleophile–electrophile distances within 3.5 Å and the *θ*_inl_ angle >140°. In the crystal structure of the product form, the A63:Cm–guanine O6 distance is 2.8 Å (suggesting these atoms are in nonbonded contact) and A63:N1–Cm–*O*^6^mG:O6 angle is 167.9°. Figure 1C shows the inline fitness landscape from the solution simulations. The simulations predict that 80.7% of reactant state trajectory frames occupy distances <3.5 Å and angles >140° (as denoted by the upper left box in Figure 1C), suggesting A63 is effectively positioned for reaction a majority of the time. Further analysis of hydrogen bonding patterns in relation to inline fitness can be found in Supplementary Figure S2. Taken together, this provides strong evidence that the dynamical ensemble described here with C10 protonated is representative of a plausible active state in solution that can be used as a departure point for further investigation of the chemical steps of the reaction using multiscale QM/MM simulations.

### Catalytic pathway for methyl transfer reaction

Having determined a plausible active state dynamical ensemble in solution, we set out to map out the free energy surface (FES) for the catalytic chemical steps of the reaction and mechanistic pathway. We consider two reaction coordinates: a methyl transfer progress variable that is a difference in the leaving group departure and nucleophilic attack distances [*ξ*_MT_ = *R*(*O*^6^mG:O6–Cm) − *R*(A63:N1–*O*^6^mG:Cm)], and a proton transfer progress variable [ξ_PT_ = *R*(C10:N3–H) − *R*(*O*^6^mG:N1–H)]. The reactant and product states have (ξ_MT_, ξ_PT_) values of approximately (−1.5, −1 Å) and (1.5, 1 Å), respectively (see Supplementary Figure S3).

We first exhaustively mapped out the full 2D FES using QM/MM simulations at the DFTB3 level (Supplementary Figure S3) and determined the existence of a low-barrier stepwise pathway (∼17.5  kcal·mol^−1^) in which proton transfer precedes methyl transfer. We also considered a high-barrier stepwise pathway (∼32.0  kcal·mol^−1^) in which methyl transfer occurs first, followed by proton transfer as well as a concerted mechanism whereby proton transfer and methyl transfer occur synchronously, and this led to single high barrier of ∼31.8  kcal·mol^−1^. Although the DFTB3 results are not expected to be quantitatively accurate, the stark difference in barriers strongly suggests that the most likely mechanism corresponds to the low-barrier stepwise path. Informed by these results, we set out to determine the minimum free energy pathway and barriers at the much more computationally intensive and quantitatively accurate *ab initio* QM/MM level.

**Figure 2.**
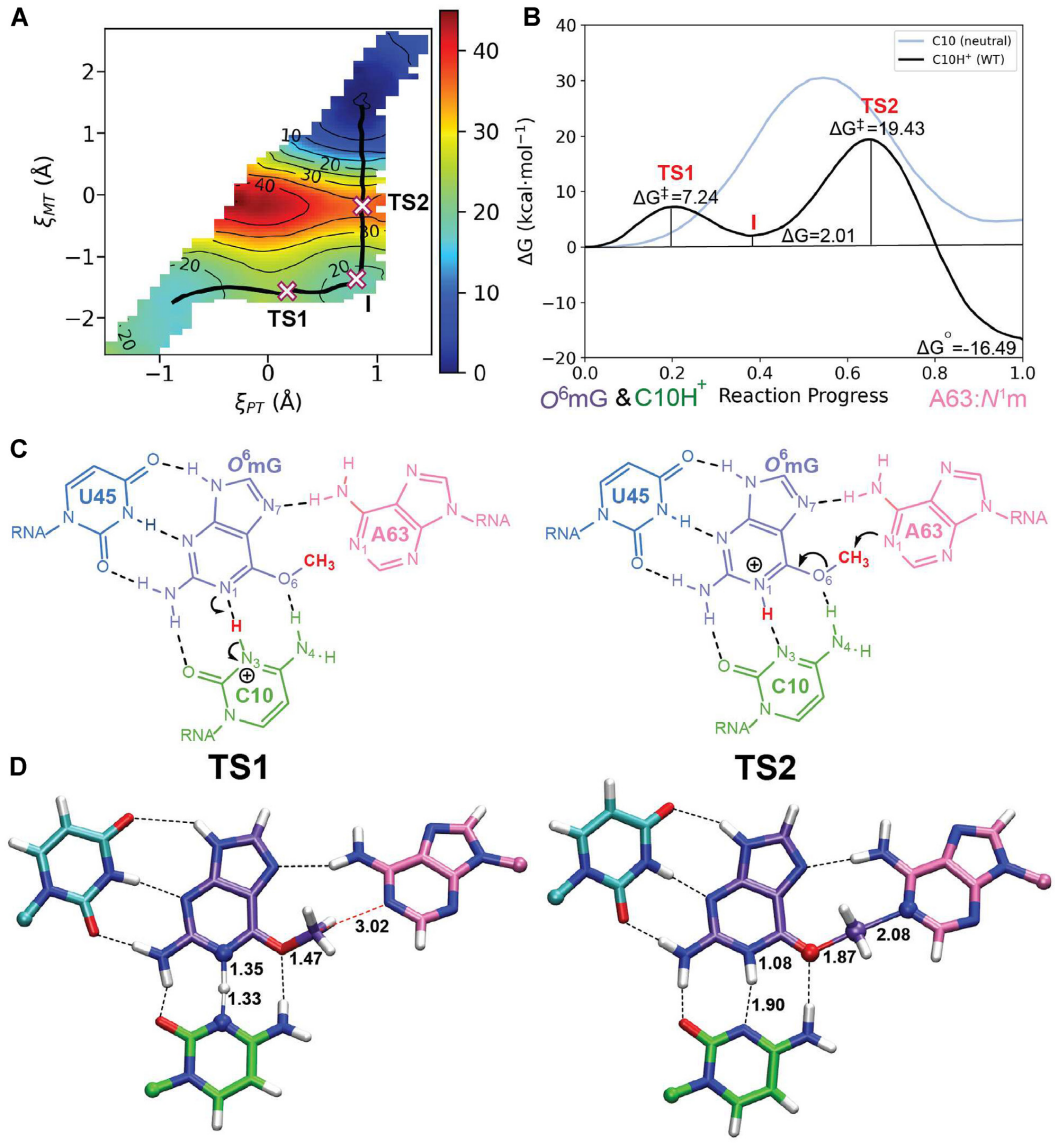
Results of *ab initio* QM/MM simulation of the reaction trajectory of MTR1 departing from the active state in solution. (**A**) 2D free energy landscape from *ab initio* QM/MM simulations with converged, stepwise path overlaid (black) and stationary points marked with crosses. (**B**) Free energy profiles (46) corresponding to the path indicated in panel (A) shown in black and resulting from the 1D reaction coordinate of methyl transfer in the absence of C10 protonation shown in gray. (**C**) Schematic of the active site depicting the transition states and corresponding mechanistic steps. (**D**) Average transition state structures calculated from additional 5 ps sampling at the stationary points found on the minimum free energy path. Bond lengths (Å) corresponding to reaction coordinates are indicated.

Toward this end, we performed *ab initio* QM/MM path simulations using the finite temperature umbrella sampling string method departing from the concerted pathway described above as an initial guess so as not to bias the search (see the ‘Detailed Computational Methods’ section in the Supplementary Data, Supplementary Figure S4 and Supplementary Table S2). The converged *ab initio* QM/MM string simulations predicted a stepwise pathway similar to that derived from the DFTB3 QM/MM simulations (Figure 2A) and, taken together with the DFTB3 results, eliminated the alternative pathways as feasible; thus, computational effort was not spent to refine this path at the *ab initio* QM/MM level. The minimum free energy pathway (Figure 2A and B) passes through two transition states, the first (TS1) corresponding to the proton transfer with a barrier of 7.2  kcal·mol^−1^ and the second rate-controlling transition state (TS2) corresponding to methyl transfer with a barrier of 19.4  kcal·mol^−1^. These transition states are illustrated mechanistically and structurally in Figure 2C and D, respectively.

The transition states are separated by a metastable intermediate (I) 2.0  kcal·mol^−1^ higher in free energy than the reactant state. The *O*^6^mG intermediate carries a positive charge and exhibits resonance to Cm that promotes *O*^6^mG:O6–Cm bond cleavage and in turn Cm–A63:N1 bond formation in the subsequent step. Considering these fundamental requirements, the nucleophilicity of adenine and ability of the ligand to stabilize positive charge are essential. This is plausibly reflected in experiments by Scheitl *et al.* in which a split (three-strand) MTR1 construct catalyzes the reaction involving *O*^6^-(*p*-aminomethyl)benzylguanine (ab^6^G) substrate more rapidly than *O*^6^mG (33). A possible cause for the increase in rate could be that ab^6^G stabilizes positive charge more effectively through resonance, thus encouraging *O*^6^–Cm bond cleavage (see Supplementary Figure S5).

In order to investigate further the active protonation state of C10 suggested by experiment, we performed *ab initio* QM/MM simulations of the reaction with neutral C10 (deprotonated at N3). In this scenario, there is not an available proton at C10:N3 to be transferred to the *O*^6^mG substrate prior to nucleophilic attack (and hence only the ξ_MT_ reaction coordinate was considered). Calculations of the 1D free energy profile predicted a pathway with a single high-barrier (30.5  kcal·mol^−1^) transition state. This barrier is >10  kcal·mol^−1^ higher than the corresponding barrier with the active state where C10 is protonated. Taken together with the calculated p*K*_a_ shift predictions described below, these results provide strong evidence that the catalytic mechanism requires C10 to be protonated in the active state.

### Reconciling crystallographic data in the context of mechanism

In the crystal structure reported by Deng *et al.* (32), the experimental distance between the *O*^6^mG:O6 and A63:N1 is 4.2 Å. The crystal structure represents the product state of the reaction in which the methyl group (Cm) has been transferred to A63:N1. Contemplating the reverse step of the reaction, given that a C–O single bond distance is ∼1.4 Å, this implies the C–N distance in the previous step should be around 2.8 Å. At this distance, however, one does not expect there to be great orbital overlap between the Cm and A63:N1 atoms required to initiate bond formation. This begs the question as to whether a local structural rearrangement from the crystal is required in order to initiate nucleophilic attack.

**Figure 3.**
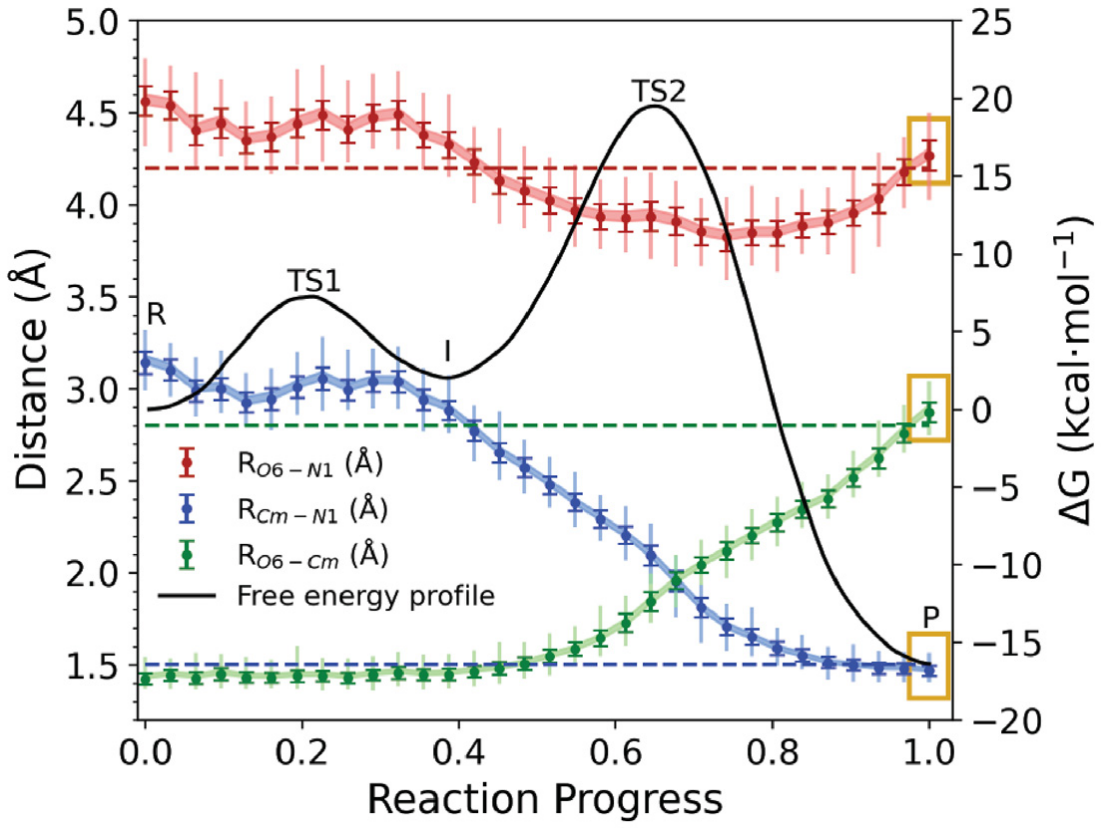
Analysis of methyl transfer distances (Å) in the context of the free energy profile (black) from *ab initio* QM/MM simulations. The distances within *O*^6^mG:O6–A63:N1 (red), Cm–A63:N1 (blue) and *O*^6^mG:O6–Cm (green) were measured for the trajectory average structures of each umbrella window on the converged reaction path. Statistical values such as 95% confidence intervals (shaded), standard deviations (dark error bars) and range of observed values (vertical lines) are traced through the trajectories for each umbrella window. The distances in the crystal structure of the product state are indicated by dashed lines.

In order to reconcile this question, we examined the progression of the *O*^6^mG:O6–A63:N1 distance, as well as the distances of the breaking/forming bonds to the transferred methyl Cm, along the reaction coordinate. These average distances, as well as their standard deviations and 95% confidence intervals, are illustrated in Figure 3 along with the computed free energy profile and dashed lines that reference the product state crystallographic distances (average distances and dynamical ranges are listed in Supplementary Table S3). The rate-controlling transition state, TS2, is associated with bringing *O*^6^mG:O6 and A63:N1 close enough such that orbital overlap between A63:N1 and Cm may initiate nucleophilic attack and chemical bonding. What is immediately clear is that in the product state (reaction progress value of 1.0), the average distances from the simulations are very close to the experimental reference values (highlighted in yellow) and in all cases the range of dynamical fluctuations encompasses the reference values. This indicates that no local rearrangement from the crystal structure is required in the above approach; rather, the distances will undergo the required adjustments as the reaction proceeds away from the minima in the profile and into the transition state region.

Departing from the intermediate, the *O*^6^mG:O6–A63:N1 distance (red) contracts from an average value of 4.32 Å to a value of 3.94 Å as it passes through the rate-controlling transition state (TS2) where it samples a range between 3.71 and 4.17 Å, and then increases again to 4.27 Å as it reaches the product. The minimum value of the *O*^6^mG:O6–A63:N1 average distance is 3.83 Å and occurs slightly after TS2 as the Cm–A63:N1 bond is almost fully formed (1.71 Å) and the *O*^6^mG:O6–Cm bond is partially broken (2.13 Å). In the intermediate, the Cm–A63:N1 distance is 2.88 Å, and in the product the *O*^6^mG:O6–Cm distance is 2.87 Å, very close to the experimental value of 2.8 Å. These values are typical of closely packed nonbonded interatomic C–N and C–O separation distances. We thus conclude that the crystal structure (32) represents a stable product state minimum consistent with simulations, and that the requisite orbital overlap for bond formation is achieved as a result of progression from stable nonbonded equilibrium states to higher free energy states along the reaction profile.

### Interpretation of apparent p*K*_a_ values in activity–pH profile for MTR1

The measurement of activity–pH profiles provides important data that report on the sensitivity of the observed rate to the protonation state (36,52,65,66). The interpretation of experimental activity–pH profiles of ribozymes is often not straightforward due to complicating factors (36,67). In order to gain mechanistic insights from these data, one must ascertain which protonation events give rise to the shape of the profiles and their maximum rates. This requires identification of nucleotides where specific protonation states are critical for function, and then to ascertain the p*K*_a_ values associated with these states in the ribozyme environment.

The experimental activity–pH profile for MTR1 is a bell-shaped curve with apparent p*K*_a_ values of 5.0 and 6.2 that were tentatively assigned to A63 and C10, although without experimental validation (32). Several factors may impact these data. Ideally, experiments would be conducted with a saturating concentration of substrate; however, the low solubility of guanine and associated compounds may preclude this. The substrate concentration used was 50 μM, but the *K*_d_ of binding is currently unknown. As the protonation of C10 may plausibly affect the affinity of substrate binding, it is possible the activity–pH profile is influenced by variable substrate affinities. Furthermore, the steep decline in activity below pH 5.5 observed by Scheitl *et al.* (33) suggests that destabilization of the ribozyme structure may influence the apparent p*K*_a_ values. These limitations of the experimental approach make direct comparison with predicted p*K*_a_ results more problematic, but at the same time highlight why these computational studies are valuable.

**Figure 4.**
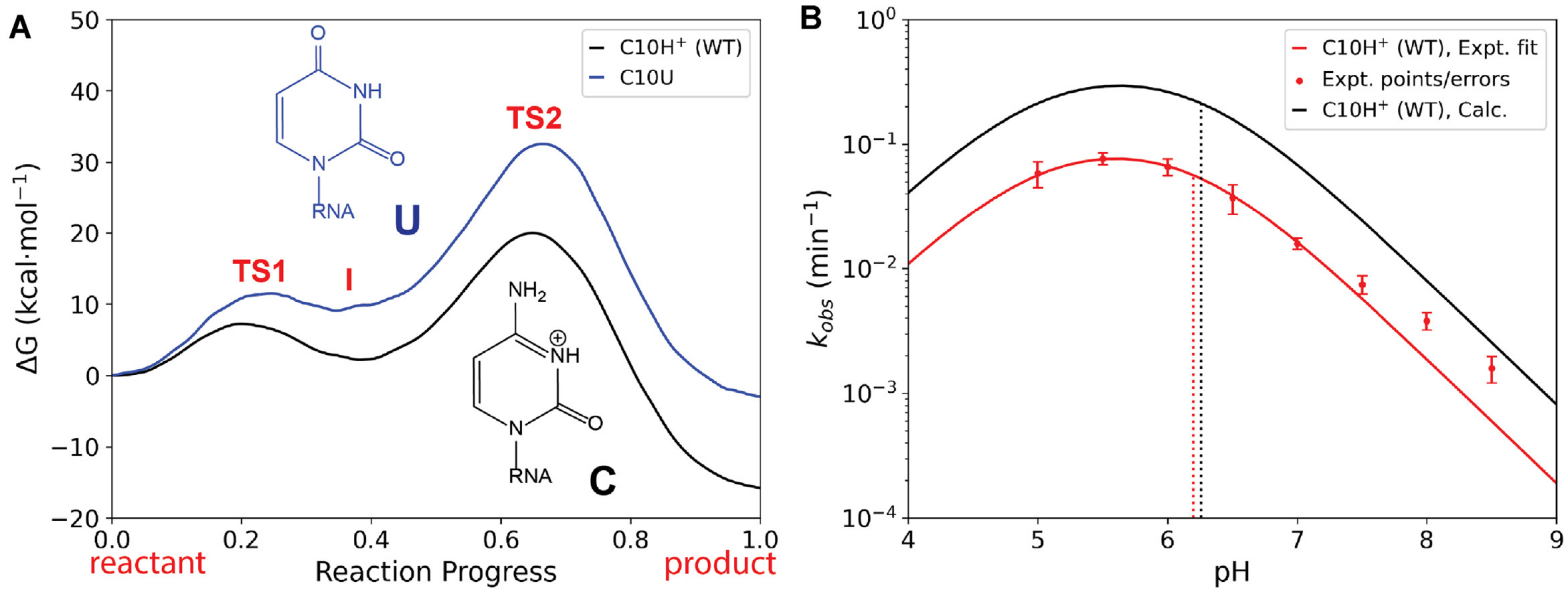
Prediction of activity–pH profiles for C10H^+^ (WT) based on the free energy barrier from QM/MM and p*K*_a_ shift from AFE simulations. (**A**) Free energy profiles corresponding to QM/MM simulations of WT C10H^+^ (black) and C10U mutation (blue). (**B**) Predicted (black) and experimental (red) WT activity–pH profile calculated using the two-state model shown in Equation (3). Red points and error bars are those of Deng *et al.* (32). Dotted lines indicate the predicted and experimental C10 p*K*_a_ values, and the lower p*K*_a_ of 5.0 from experiment is assumed. The calculated C10U mutant results were below the detection limit of 10^−6^ min^−1^ (in accord with experiment) and hence not visible on the scale of the plot. Computational error analysis of the free energy profiles is given in Supplementary Figure S6.

The purpose of this section is to provide computational support through QM/MM and AFE simulations to aid in the interpretation of the measured activity–pH profiles, and in particular by providing support for C10 as the general acid apparent p*K*_a_ value (32,33). Given the experimental p*K*_a_ of *O*^6^mG is quite low (2.35) (68), we eliminated the substrate as a likely candidate to account for either of the observed apparent p*K*_a_ values of 5.0 or 6.2. In the previous section, *ab initio* QM/MM simulations predicted the protonation of C10 to be required for catalysis, implicating this nucleotide as a possible candidate for the acid apparent p*K*_a_. To test this hypothesis further, we performed additional *ab initio* QM/MM simulations of the C10U mutant. Uracil has a p*K*_a_ value at the N3 position of ∼9.2, upshifted from that of cytosine by 5 units, suggesting it will be much more difficult to abstract a proton from this position (69). The QM/MM simulations (Figure 4A) predict a rate-controlling barrier of 32.6  kcal·mol^−1^, slightly higher than that predicted for neutral C10 with N3 deprotonated (Table 1). This barrier corresponds to an intrinsic rate constant of ≈10^−11^ min^−1^, which is well below an experimental detection limit of 10^−6^ min^−1^, in agreement with the severely impaired activity observed experimentally for this mutation (32). We thus sought to determine the p*K*_a_ value of C10:N3 to ascertain the degree to which it corresponded to the experimentally measured apparent p*K*_a_.

**Table 1.** Free energy profile data from QM/MM simulations of the WT and C10U mutation with predicted cleavage rates^a^

	Free energy (kcal·mol^−1^)				Rate (min^−1^)	
Species	$\Delta G_{{\rm TS}1}^{\ddagger }$	Δ*G*_I_	$\Delta G_{{\rm TS}2}^{\ddagger }$	Δ*G*_rxn_	*k*_int_(calc.)	*k*_int_(expt.)
C10H^+^ (WT)	7.24(22)	2.01(24)	19.43(17)	−16.49(07)	0.45(06)	0.12
C10 (neutral)	N/A	N/A	30.53(14)	4.61(27)	≈10^−8^	N/A
C10U	11.56(35)	9.13(43)	32.59(49)	−2.87(12)	≈10^−11^	<10^−6^

^a^Free energy values (kcal·mol^−1^) for stationary points in the free energy profiles shown in Figures 2B and 4A. Intrinsic reaction rates (min^−1^) were estimated from Equation (S1) in the Supplementary Data, where Δ*G*^‡^ is the rate-controlling transition state free energy barrier listed in this table and shown in Figure 4. The intrinsic rate for the C10U mutant is below the experimental detection limit of <10^−6^ min^−1^. Since the experimental intrinsic rate is taken to result from C10H^+^, the C10 (neutral) experimental rate is not applicable (N/A). Standard error estimates are given in parentheses multiplied by 100, and error bars for the calculated profiles shown in Figure 4B are provided in Supplementary Figure S6.

We performed AFE simulations to calculate the microscopic p*K*_a_ shift of C10 in the substrate-bound form of MTR1 according to the transformations shown in Supplementary Figure S7. Results are listed in Table 2. The value of the acid shift relative to the solution value of 4.2 (55) was determined to be 2.06 when *O*^6^mG is bound, leading to a predicted p*K*_a_ value of 6.26 in MTR1, very close to the apparent p*K*_a_ value of 6.2 observed experimentally. This p*K*_a_ prediction, together with the computational evidence of the preceding section demonstrating the critical role of C10 protonation in the catalytic mechanism, provides strong support for the original experimental supposition (32) that C10 is the most likely candidate from which the apparent p*K*_a_ of 6.2 arises. The upshifted p*K*_a_ of C10 can be explained by examination of the strong and persistent hydrogen bond with the *O*^6^mG:N1 position, and thus protonation of C10 is likely correlated with substrate binding.

**Table 2.** p*K*_a_ values and shifts of the predicted general acid (C10:N3) and nucleophile (A63:N1) nucleotides in the ligand-bound form of MTR1^a^

		Soln.	MTR1 (bound)		Expt.
Nucleotide	Role	p*K*_a_	Δp*K*_a_	p*K*_a_	App. p*K*_a_
C10	General acid	4.2	2.06(03)	**6.26**	**6.2**
A63	Nucleophile	3.5	−0.74(62)	2.76	**5.0**

^a^Shifts are calculated relative to experimental solution values (55,56) (Soln.) from AFE simulations for MTR1 bound to the *O*^6^mG substrate. The experimental apparent p*K*_a_ values are shown for reference; however, the respective roles were not experimentally validated. Values shown in bold are parameters used to construct the activity-pH profiles in Figure 4. Analysis of enhanced sampling for AFE simulations used to determine the p*K*_a_ shifts is provided in Supplementary Table S5 and Supplementary Figure S8.

The origin of the lower apparent p*K*_a_ value of 5.0, however, was unclear from experiment. Several naturally occurring ribozymes that use a general acid–base strategy for catalysis exhibit bell-shaped activity–pH profiles similar to that of MTR1. Experiments and computation indicate MTR1 employs a general acid, but not a general base. Nonetheless, the A63:N1 position is the nucleophile in the reaction (i.e. acts as a Lewis base), and thus would be functionally sensitive to and essentially inactivated by protonation at N1. We thus considered A63:N1 as a conceivable candidate as the origin of the unidentified apparent p*K*_a_. Computing the p*K*_a_ shift of A63 relative to the solution value of 3.5 (56) in the bound form of the ribozyme results in downshifting by 0.74 p*K*_a_ units due to the proximity of the *O*^6^mG methyl group (Table 2). The resulting p*K*_a_ value of 2.76 is too low to significantly influence the experimentally observed activity–pH profile, leading us to conclude that the lower apparent p*K*_a_ of 5.0 does not arise from protonation of A63:N1.

Alternative explanations for the apparent p*K*_a_ of 5.0 could include protonation of one or more of A9, A40 or A46, which form a base triple that stacks with the ligand binding nucleotides; however, it is unclear how these protonation events may impact catalysis. Alternatively, the decrease in activity at low pH may arise from destabilization of the ribozyme or weaker substrate binding.

The AFE simulations thus strongly implicate C10:N3 as the origin of apparent p*K*_a_ of 6.2, but do not support the hypothesis that A63:N1 may be the origin of the apparent p*K*_a_ of 5.0. Based on the calculated p*K*_a_ values, we sought to predict the full activity–pH profile from computations such that direct comparison could be made with the experimental profile.

In order to predict the full activity–pH profile from Equation (3), in addition to the computed p*K*_a_ values (Table 2), one must determine the intrinsic rate (*k*_int_), which can be obtained from *ab initio* QM/MM simulations [see Equation (S1) of the Supplementary Data]. The calculated free energy values and estimated intrinsic rates are listed in Table 1, and the resulting computed activity–pH profile is illustrated in Figure 4B. To our knowledge, these results represent one of the first reported activity–pH profiles predicted from a combination of *ab initio* QM/MM and AFE simulations. The calculated and measured profiles are very similar, apart from the divergence at low pH, and are shifted by roughly half of a log unit. This agreement is very encouraging and provides support for the validity of the simulation results and their interpretation.

### Mechanism of MTR1 bears similarity to that predicted for *O*^6^-alkylguanine-DNA alkyltransferase protein

The observation of methyl and alkyl transfer activity by RNAs provides support for the RNA world hypothesis by demonstrating that the limited RNA library can include molecules that are capable of diverse chemistry similar to that observed in naturally occurring protein enzymes. Parallel mechanistic concepts emerge from study of *O*^6^-alkylguanine-DNA alkyltransferase (AGT) protein that directly repairs potentially carcinogenic *O*^6^-methylguanine bases in DNA (70). The mechanism of AGT has been studied previously using QM optimization methods on an active site cluster model (71) that suggested a stepwise mechanism involving nucleophile (Cys145) activation through His146 that acts as a water-mediated general base, followed by nucleophilic attack to dealkylate the guanine base. In a later study, QM/MM calculations (nondynamical path optimization) in a spherical partially frozen water droplet (72) supported a mechanism where AGT acts in a stepwise manner of nucleophile (Cys145) activation, followed by methyl transfer that occurs as a two-step migration with a stable intermediate (72). These simulations predicted a rate-controlling barrier of 18.2  kcal·mol^−1^ for the first step of the methyl transfer and differed from the previous cluster model work that predicted the methyl transfer to occur in a single step with higher rate-controlling barrier (23.2  kcal·mol^−1^). In the present work, we performed *ab initio* QM/MM simulations of a fully explicitly solvated MTR1 system under periodic boundary conditions (45) to determine the free energy profile and pathway. Despite methodological differences between these works, the common theme is that MTR1 and AGT likely exhibit similar catalytic mechanisms (i.e. stepwise mechanisms with rate-controlling methyl transfer steps), highlighting an intriguing connection between these RNA and protein enzymes.

## CONCLUSION

Here, we have used a combination of classical MD, QM/MM and AFE simulations to explore the mechanism of methyl transfer from *O*^6^mG to N1 of a specific adenine in the MTR1 ribozyme. The structure and dynamics in solution involve only modest relaxation from the crystal and support an active state in which C10, protonated at the N3 position, forms a key hydrogen bond with the N1 position of the *O*^6^mG substrate. The lowest energy reaction pathway determined by QM/MM free energy simulations suggests a stepwise mechanism that passes through two transition states separated by a metastable intermediate, as opposed to a concerted mechanism. The first transition state (7.2  kcal·mol^−1^ barrier) corresponds to the proton transfer from C10:N3 to *O*^6^mG:N1, whereas the second rate-controlling transition state corresponds to methyl transfer (19.4  kcal·mol^−1^ barrier). Simulations of the reaction with neutral C10 led to a barrier >10  kcal·mol^−1^ higher, highlighting the critical role of this residue acting as an acid in catalysis. In order to aid in the interpretation of experimental activity–pH profiles, AFE simulations predicted the p*K*_a_ for C10 to be 6.3, very close to the experimental apparent p*K*_a_ of 6.2, strongly implicating this residue as a critical acid, in support of the QM/MM simulation results. Finally, we make mechanistic comparison of MTR1 with AGT. MTR1 has evolved *in vitro* for alkyl transfer activity as would be required for the metabolism of a putative RNA world. It is thus of considerable interest that there are clear mechanistic similarities to naturally occurring protein enzymes like AGT. The results of the current work provide further support for evolutionary theories that suggest life originated in an RNA world, and help to establish design frameworks for new RNA-based biochemical tools.

## DATA AVAILABILITY

The data underlying this article will be shared on reasonable request to the corresponding author.

## Supplementary Material

gkad260_Supplemental_File
