# Supplementary material for: Catalytic mechanism and pH dependence of a methyltransferase ribozyme (MTR1) from computational enzymology
Source: Nucleic Acids Res. 2023 Apr 18;51(9):4508–18. doi: 10.1093/nar/gkad260 (PMC10201425; doi:10.1093/nar/gkad260)
Supplement: gkad260_Supplemental_File [file gkad260_supplemental_file.pdf]

Catalytic mechanism and pH-dependence of a methyl transferase ribozyme (MTR1) from computational enzymology

Erika McCarthy[1] , Şölen Ekesan[1], Timothy J. Giese[1], Timothy J. Wilson[2], Jie Deng[3], Lin Huang[3], David M. J. Lilley[2], Darrin M. York [1,*]

[1] *Laboratory for Biomolecular Simulation Research, Institute for Quantitative Biomedicine and Department of Chemistry and Chemical Biology, Rutgers University, Piscataway, NJ 08854, USA*
[2] *Nucleic Acid Structure Research Group, MSI/WTB Complex, The University of Dundee, Dundee, UK*
[3] *Guangdong Provincial Key Laboratory of Malignant Tumor Epigenetics and Gene Regulation, Guangdong-Hong Kong Joint Laboratory for RNA Medicine, Sun Yat-sen Memorial Hospital, Sun Yat-sen University, Guangzhou 510120, China; Medical Research Center, Sun Yat-sen Memorial Hospital, Sun Yat-sen University, Guangzhou 510120, China.*
Email: Darrin.York@rutgers.edu

SUPPLEMENTARY DATA

Detailed Computational Methods

Molecular Mechanical MD Simulations

All molecular mechanical (MM) simulations were carried out with the PMEMD program included in the AMBER22 software package (1). The parameters were taken from the ff99OL3 (2, 3) RNA force field (4), and the O^6mG ligand was parameterized using the Antechamber program (5), which assigned AM1-BCC charges (6) and GAFF force field (7) parameters. The system was prepared by solvating the MTR1 (PDB ID 7V9E (8)) crystal structure coordinates in a truncated octahedron (90.2 Å real-space lattice vector lengths) with 18250 TIP4P-Ew water molecules (9), 133 Na^+, and 47 Cl^- ions to neutralize the ribozyme charge and achieve a physiological ion concentration of 140 mM NaCl. We used the monovalent ion parameters designed for use with the TIP4P-Ew water model (10). Electrostatics were performed with the particle mesh Ewald method (11, 12) using 12 Å real-space cutoffs, a 1 Å^3 regular grid, and tinfoil boundary conditions. All simulations were carried out with a 1 fs time step, and the SHAKE algorithm (13) was used to fix hydrogen bond lengths.

The system was prepared in a series of steps which slowly increased the temperature and equilibrated the density while restraining the solute atoms' positions to the crystal structure coordinates. This was followed by a second series of simulations that slowly removed the restraining potentials. First, the energy was minimized with the conjugate gradient method for 500 steps while restraining the solute coordinates with 50 kcal$\cdot$ mol$^{-1}\cdot$Å^{-2} force constants. The temperature of the restrained system was increased at constant volume from 0 K to 300 K over a period of 600 ps, and the simulation at 300 K was extended for an additional 1 ns. The density of the restrained system was then equilibrated in the isothermal-isobaric ensemble at 300 K and 1 atm using the Berendsen barostat (14). The restraint force constants were then reduced to 25 kcal$\cdot$ mol$^{-1}\cdot$Å^{-2}, and 2000 steps of conjugate gradient method was performed. The system was then heated from 0 K to 300 K over a period of 1.6 ns at constant volume. Finally, 2 ns of simulation was performed in the isothermal-isobaric ensemble at 300 K and 1 atm during which the restraint force constants were reduced from 25 kcal$\cdot$mol$^{-1}\cdot$Å^{-2} to 0 kcal$\cdot$mol$^{-1}\cdot$Å^{-2}.

To analyze the results and estimate statistical uncertainties, we carried out 5 production simulations initiated from different starting structures. Each production simulation was run for 150 ns in the isothermal-isobaric ensemble at 300 K and 1 atm. The first production simulation began from the coordinates obtained from the system preparation described above. Analysis of the solute coordinate root-mean-square deviation (RMSD) with respect to the starting structure (see Figure S1) suggests that the first 50 ns should be excluded as equilibration. To obtain initial structures for the remaining 4 production simulations, we extracted the coordinates from the first trial at 10 ns, 15 ns, 20 ns, and 25 ns. Each of the remaining 4 trials were repeated for 150 ns and the first 50 ns was excluded as equilibration.

QM/MM Simulations

The DFTB3 QM/MM simulations of the wild-type ribozyme departed from the equilibrated reactant state structure described in the previous section. The QM region has a net 1+ charge, and it includes 45 atoms consisting of the ligand and the C10 and A63 nucleobases. The QM/MM boundary at the C10 and A63 glycosidic bonds was treated with the link-atom approach (15, 16). The $\xi_{\rm PT}$ and $\xi_{\rm MT}$ umbrella potentials were simulated with 100 kcal$\cdot$mol^{-1}Å^{-2} force constants. Several half-harmonic biasing potentials were included to ensure that the hydrogen bond network was maintained when high-energy regions of the FES were sampled. The additional restraints used one-sided harmonics (with 100 kcal$\cdot$mol^{-1}Å^{-2} force constants) to prevent the following hydrogen bonds from exceeding the following values: U45:O2-O^6mG:H4 (2.2 Å), U45:H3-O^6mG:N3 (2.2 Å), U45:H42-O^6mG:O (2.2 Å), A63:H61-O^6mG:N (3.0 Å), C10:O4-O^6mG:H6 (3.0 Å), and C10:O2-O^6mG:H5 (2.2 Å). The DFTB3 QM/MM simulations were performed for 12.5 ps with a

1 fs time step in the canonical ensemble at 298 K. The long-range electrostatics were evaluated using the Mulliken charges, a 10 Å real-space cutoff, a 1 $Å^3$ regular grid, and tinfoil boundary conditions (17). The FES was generated from the analysis of the last 10 ps of each simulation using the multistate Bennett acceptance ratio method (18, 19) implemented in the ndfes program available within the FE-ToolKit software package (20).

The mechanism of the adenine N1-methylation reaction in the wild-type ribozyme was examined with PBE0/6-31G* QM/MM simulation using the finite temperature string method. The initial string departed from the concerted pathway connecting the reactant and product states using 32 equally spaced windows and 100 kcal·mol^{-1}Å^{-2} force constants. Each string iteration consisted of 1 ps/window sampling in the canonical ensemble at 298 K with PBE0/6-31G* QM/MM and a 1 fs time step. The ambient-potential composite Ewald method (21) was used to treat long-range electrostatics with a 10 Å real-space cutoff, a 1 $Å^3$ regular grid, and tinfoil boundary conditions. A total of 40 string iterations were performed to converge the pathway. The 32 windows along the final pathway were then simulated for 5 ps, and the simulations were performed 4 times with different thermostat random number seed values to estimate errors in the final free energy values.

The procedure for calculating the DFTB3 2D surface of the C10U ribozyme mutant is analogous to the wild-type simulations described above. Because the 2D surfaces yield similar minimum free energy pathways, the PBE0/6-31G* QM/MM string calculations of the mutant ribozyme was initiated from the wild-type's converged pathway. The pathway was refined using 5 additional string iterations, each string consisted of 32 windows, and each simulation was run for 1 ps. The final strings were then sampled for an additional 5 ps.

We estimate the intrinsic rate constant from transition state theory, where the activation free energy is taken from the PBE0/6-31G* QM/MM profiles described in the previous section.

$$k_{\text{int}} = \kappa \frac{k_B T}{h} e^{-\frac{\Delta G^\ddagger}{k_B T}}, \tag{S1}$$

h is Planck's constant, k_B is the Boltzmann constant, $T = 298$ K is the temperature, and κ is a transmission coefficient. We used the reactive flux correlation function approach (22–24) to estimate a transmission coefficient from DFTB3 simulations to reduce computational cost. To estimate the DFTB3 transmission coefficient, we performed 32 unbiased simulations of 64 initial structures (2048 net simulations) representing the transition state. The initial velocities were randomized to a Maxwell-Boltzmann distribution, the dynamics were performed for 50 ps with a 0.5 fs time step, and a time-dependent transmission coefficient was computed by following the procedure described in ref. 25. The time-dependent transmission coefficient reaches a plateau value, which is taken to be DFTB3 transmission coefficient κ_{DFTB3}. This value is then corrected to estimate the *ab initio* transmission coefficient κ from Kramer's theory in the high friction limit (26). In this limit, the transmission coefficient $\kappa = \omega^\ddagger/\gamma$ is related to the imaginary frequency of the transition state $\omega^\ddagger$ and a friction coefficient γ. If we assume that the friction coefficient is the same at the DFTB3 and *ab initio* levels of theory, then one obtains the approximation:

$$\kappa = \frac{\omega^\ddagger_{\text{PBE0}}}{\omega^\ddagger_{\text{DFTB3}}} \kappa_{\text{DFTB3}} \tag{S2}$$

The procedure described above yields $\kappa = 0.214$ for the wild-type reaction. The activation energy of the C10U reaction is so high that the rate estimated from conventional transition state theory is several orders of magnitude smaller than the experimental detection limit; in this case, we do not compute κ. Finally, standard errors of the free energies computed using MBAR are estimated by the ndfes program (20) by the propagation of bootstrap errors.

Alchemical Free Energy Simulations

All alchemical free energy (AFE) simulations were performed with the AMBER Drug Discovery BOOST package (27), and the simulations were prepared using an automated work-flow originally designed to calculate protein-ligand relative binding free energies (28), which we have adapted for use to evaluate deprotonation free energies. The force field parameters for the protonated C10 and protonated A63 nucleotides were prepared, and the structure of the protonated ($\lambda = 0$) and deprotonated ($\lambda = 1$) states were rebuilt departing from the coordinates extracted from the previously described MM production simulations of the protonated solution-phase models. Each system was re-solvated and equilibrated with 15642 TIP4P-Ew waters (9), 107 Na^+, and 41 Cl^- in a rectangular box. The equilibration procedure included an initial minimization of the system with heavy-atom Cartesian restraints, and an additional 4.5 ns of simulation which increased the temperature to 300 K, equilibrated the density at 1 atm, and slowly removed the heavy atom restraints. The 2 ligand bound states were then equilibrated for an additional 50 ns in the isothermal-isobaric ensemble without restraints.

The recently developed smoothstep softcore potentials (29) were used to create intermediate states that connect the protonated and deprotonated systems. The potential energies of the C10 nucleotide are linearly transformed between the $\lambda = 0$ and $\lambda = 1$ states, and the acidic proton is included in a nonlinear softcore region. Furthermore, the $O\mathrm{CH}_3$ group on the O^6mG ligand was included in the softcore region using an approach called AlChemical Enhanced Sampling (ACES) (27, 30). The parameters of O^6mG do not differ between the $\lambda = 0$ and $\lambda = 1$ states, so this inclusion does not directly alter the estimated free energy; however, it does enhance the conformational sampling of the intermediate states. The simulation schedule was chosen to uniformly sample the second order smoothstep function (S2) with 25 windows: (31) $\lambda \in$ [0.0, 0.176834, 0.229764, 0.269379, 0.302697, 0.33229, 0.359436, 0.384886, 0.40913, 0.432518, 0.455318, 0.477748, 0.5, 0.522252, 0.544682, 0.567482, 0.59087, 0.615114, 0.640564, 0.66771, 0.697303, 0.730621, 0.770236, 0.823166, 1.0]. The intermediate states were equilibrated in sequence from the nearest end-state with 3.5 ns of simulation in the isothermal-isobaric ensemble at 300 K and 1 atm. Production statistics were gathered for 5 ns/state, and Hamiltonian replica exchange (32–35) was employed to enhance sampling using a 20 ps exchange-attempt rate. The production sampling was performed 5 times with different thermostat random number seeds to estimate errors, yielding 625 ns of aggregate production sampling. Free energies were evaluated using the MBAR method and standard errors were obtained by propagation of bootstrap errors.

The deprotonation free energy estimates in aqueous environment were performed similarly. The solute structure is a ACC or GAG RNA 3-mer sequence, whose central nucleotide represents C10 or A63 respectively. The solute is solvated in a rectangular box with a 20 Å buffer of waters and physiological concentration of monovalent ions.

Convergence of MD simulations: Root mean square deviation analysis

In order to characterize the dynamical ensemble of the active, reactant state of MTR1 in solution we conducted 5 independent MD simulations as described in the Detailed Computational Methods section. Convergence of these simulations was assessed by analyzing the root mean square deviation (RMSD) between average structures from each simulation, as well as RMSD with respect to the first frame of the trajectory. RMSD analysis over time indicates that the simulations reached convergence after ∼50 ns, therefore the first 50 ns were discarded as equilibration and further analysis was done on the final 100 ns. For each of the 5 simulations (sim 1 through sim 5) the maximum RMSD of the average structure occurs between the simulation and the crystal, indicating an expected systematic structural drift induced by solvation. In addition, the maximum RMSD between average structures of trajectories is 2.8 Å, indicating good agreement between the independent simulations and corroboration of the solution active state for further simulation. Sim 1 departed from the crystal structure and provided departure points for the subsequent simulations; however, the RMSD from the crystal of sim 1 (3.59 Å) is an outlier. The positions of heavy atoms in this simulation may have experienced the greatest amount of positional drift compared to the crystal, but it experienced similar per residue macroscopic fluctuations as the other 4 simulations. Removing this simulation from the per residue root mean square fluctuation (RMSF) analysis reduced the correlation coefficient between the experimental and simulated curve by only 0.004, suggesting the macroscopic fluctuations of the 5 simulations are in agreeance.

Table S1. RMSD (Å) of average structures of 5 independent 100 ns trajectories in the reference frame of the crystal structure.[a]

RMSD (Å)	Active site					
All heavy atoms	crystal	sim 1	sim 2	sim 3	sim 4	sim 5
crystal	-	0.88	0.69	0.68	0.63	0.65
sim 1	3.59	-	0.43	0.33	0.53	0.75
sim 2	2.69	1.84	-	0.25	0.30	0.50
sim 3	2.31	2.26	1.98	-	0.33	0.49
sim 4	2.83	2.11	1.95	1.89	-	0.42
sim 5	2.80	2.31	2.09	1.84	1.40	-

[a]RMSD values are given between the average structure of the simulations in the corresponding row and column. Values to the left of the diagonal correspond to all heavy atoms, and values to the right of the diagonal correspond to heavy atoms in the active site.

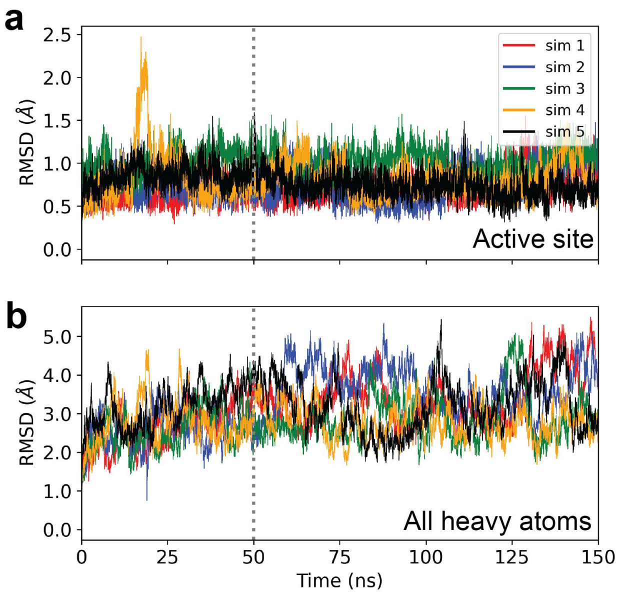

Figure S1. Root mean square deviation (RMSD) of heavy atoms to first frame of 5 independent trials. **(a)** RMSD for active site heavy atoms (C10, U45, A63, and O^6mG). **(b)** RMSD for all heavy atoms. The dashed vertical line at 50 ns indicates the point before which the trajectory data was discarded in the analysis, and after which the RMSD was observed to fluctuate stably and data (50-150 ns) was used in the analysis.

Extended analysis of inline attack

Catalytic fitness of the proposed dynamical solution ensemble was assessed by analyzing the distribution of the nucleophile-electrophile angle (O^6mG:O6-Cm-A63:N1) and distance (O^6mG:Cm-A63:N1) as shown in Figure 1c of the main text. Each trajectory frame was assigned a combined inline fitness score according to the following equations:

$$\text{score} = \frac{\text{value} - \text{cutoff}}{\text{best} - \text{cutoff}} \tag{S3}$$

$$\text{combined score} = \sqrt{\frac{(\text{angle score})^2 + (\text{distance score})^2}{2}}, \tag{S4}$$

where the best angle is taken to be 170° with a cutoff of 140°, and the best distance is taken to be 3.0 Å with a cutoff of 3.5 Å (36, 37). Scores thus ranged from 0 to 1, with trajectory frames below the cutoffs being assigned a score of 0. Colors in Figure 1c were mapped onto points based on a trajectory frame's score where a score of 1 was colored red and a score of zero was colored blue with intermediate scores transitioning from blue to red. This led to the question of whether lack of in-line fitness was simply due to random, dynamic movements or loss of an important hydrogen-bonding interaction.

Impact of hydrogen bonding on inline attack

In the crystal structure and our simulations there is a persistent hydrogen bond between O^6mG:N7 and the A63:N6 exocyclic hydrogen (H61). We analyzed this hydrogen bond distance in both catalytically active and inactive frames as defined by Figure 1c of the main text. Both distributions favor hydrogen bonding (distance less than 2.5 Å), and it does not appear that loss of this interaction is the driving force for loss of catalytic fitness; however, the existence of the N6-amino group is essential. Experiment has demonstrated that purine and 2-aminopurine substitutions at the A63 position are not active within 5 hours, further suggesting that the N6-amino group provides an essential hydrogen bond for in-line attack (38).

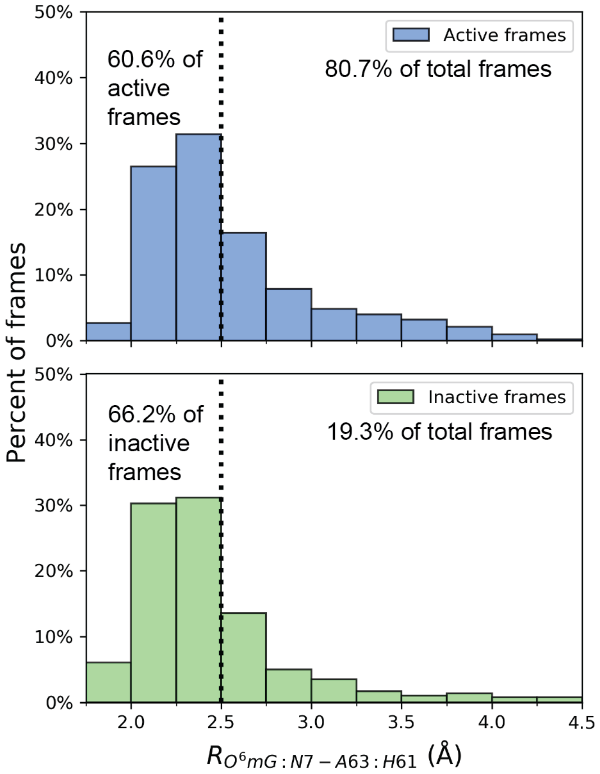

Figure S2. Distribution of distance between A63:H61 and O^6mG:N7 in active and inactive trajectory frames as shown in Figure 1 of the main text.

Semi-empirical QM/MM simulations

After determining a plausible active state ensemble, we used QM/MM simulations to map out the free energy surface corresponding to the mechanistic pathway of both the wild-type ribozyme and C10U mutant. To determine a reasonable departure point for *ab initio* QM/MM simulations, the semi-empirical DFTB3 method was used to map a 2D grid of progress coordinates as shown in Figure S3. Comparing the three potential paths gleaned from the semi-empirical grid, the pathway in which proton transfer precedes methyl transfer is more favorable by ~14.5 kcal·mol^{-1} compared to the other two paths which pass through a barrier of ~32.0 kcal·mol^{-1}. The C10U mutated system follows a similar mechanistic pathway to the wild-type but with a higher barrier (~22.3 kcal·mol^{-1}).

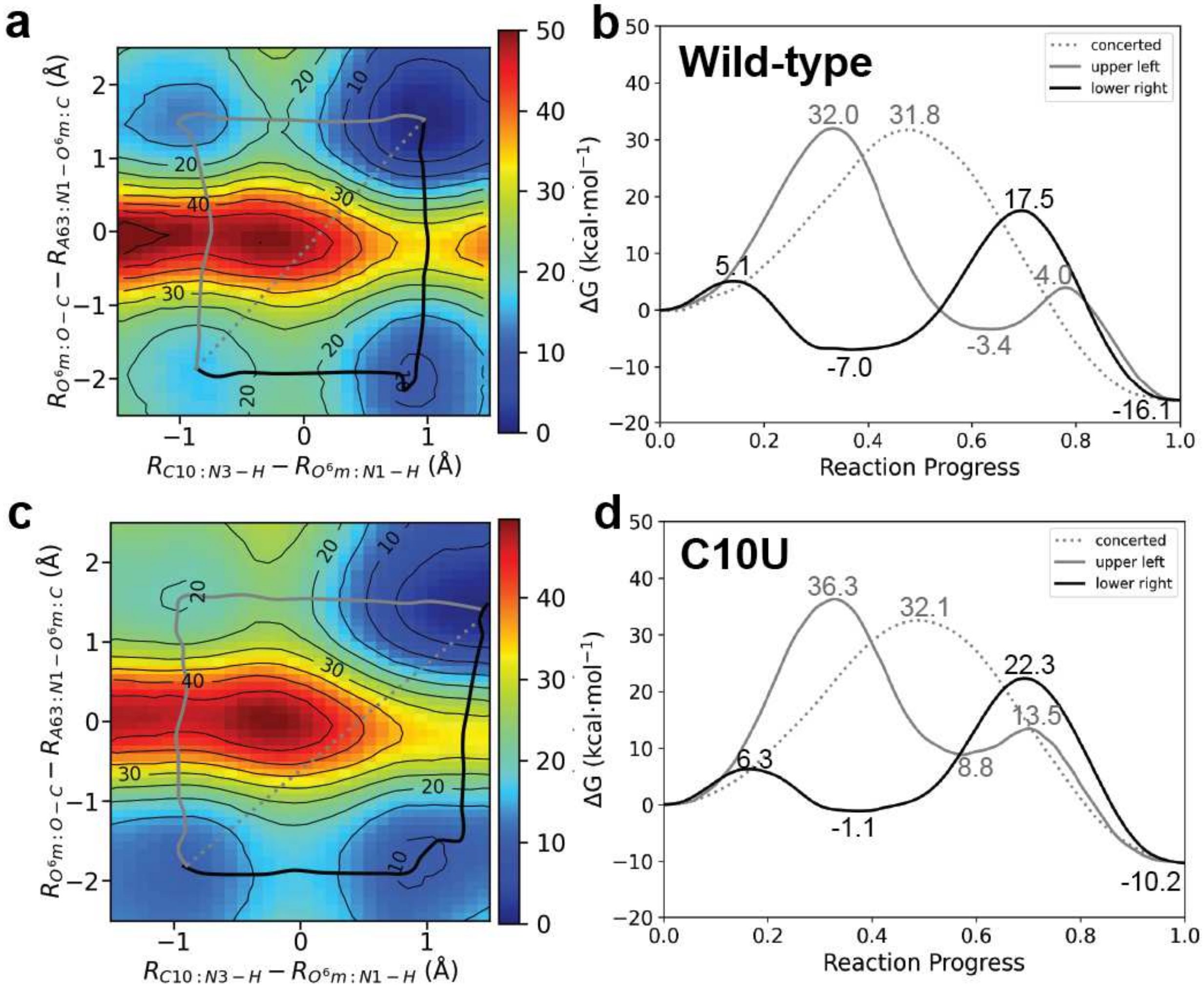

Figure S3. Semi-empirical free energy landscape of wild-type and C10U mutant reactions. **(a)** 2D wild-type DFTB3 free energy landscape with the minimum free energy path denoted in black, an alternate step-wise pathway denoted in solid gray, and a concerted pathway denoted with a gray dashed line. **(b)** 1D free energy profiles corresponding to the black and gray paths in (a). **(c)** 2D C10U mutant DFTB3 free energy landscape with minimum free energy path denoted in black and alternate pathways denoted in gray. **(d)** 1D free energy profiles corresponding to the black and gray paths in (c).

Ab initio reaction pathways

The favorable lower right pathway gleaned from DFTB3 simulations was further corroborated when wild-type PBE0 strings were initiated from the concerted path and converged upon the lower right path, ensuring that the less favorable pathway was not wrongfully ruled out. The chemical mechanism is defined by the values of the reaction coordinates that yield the minimum free energy pathway. *Ab initio* finite temperature string simulations of the wild-type ribozyme are shown in Figure 2 of the main text, and the minimum free energy path served as a departure point for *ab initio* simulations of the C10U mutant. These pathways are compared in Figure S4, and the specific location of stationary points along the reaction pathways for the simulated species is reported in Table S2. The transition state corresponding to proton transfer (TS1) in the C10U mutant occurs later compared to that of the wild-type reflecting a higher barrier of proton transfer in agreement with the higher pK_a of uracil (9.2) compared to cytosine (4.2).

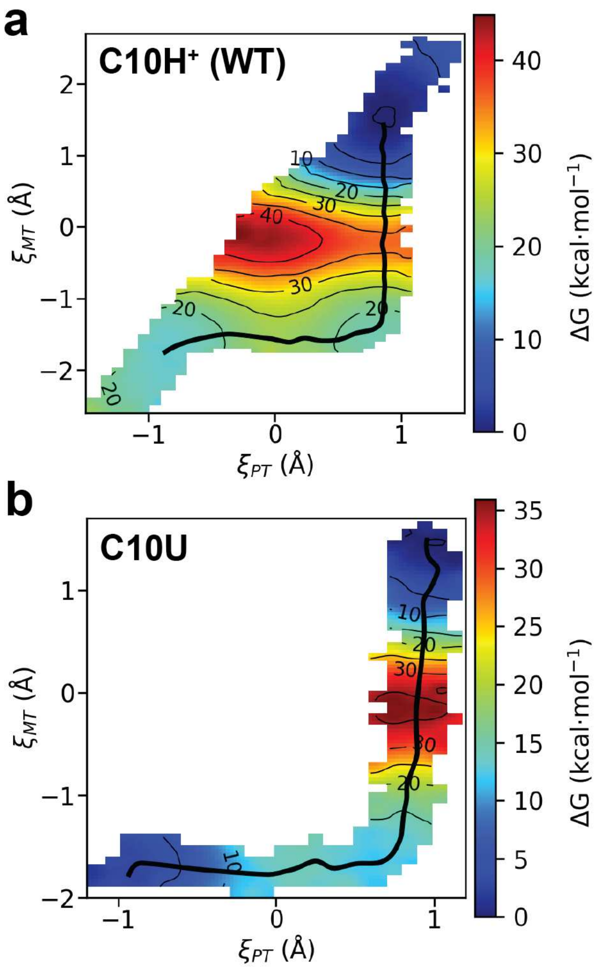

Figure S4. *Ab initio* free energy surfaces obtained using the finite temperature string method for **(a)** the wild-type (WT) ribozyme protonated at C10 and **(b)** the C10U mutant. The minimum free energy pathways are outlined in black. Simulations for C10U departed from the minimum free energy path of the wild-type.

Table S2. Reaction coordinates (Å) of key species along the reaction pathway.[a]

	C10H$^+$ (WT)		C10U	
Species	ξ_{PT}[b]	ξ_{MT}[c]	ξ_{PT}	ξ_{MT}
R	-0.87	-1.75	-0.93	-1.77
TS1	0.03	-1.59	0.19	-1.67
I	0.75	-1.45	0.62	-1.66
TS2	0.85	-0.21	0.89	-0.15
P	0.86	1.42	0.96	1.49

[a] Values of reaction coordinates for stationary points are calculated from the 2D *ab initio* surfaces in Figure S4 where ξ_{PT}=R(C10:N3-H)-R(O^6mG:N1-H) and ξ_{MT}=R(O^6mG:O6-Cm)-R(A63:N1-O^6mG:Cm).

The active site of MTR1 facilitates nucleophilic attack

Both semi-empirical and *ab initio* level simulations of the reaction pathway indicate the existence of a stable intermediate in which a proton has been transferred from C10:N3 to O^6mG:N1. The *ab initio* intermediate structure was approximately observed at ξ_{MT}, $\xi_{PT} = (-1.5, 1)$ and involves O^6mG having positive charge, which is stabilized by resonance to Cm. Favoring resonance to Cm is expected to promote cleavage of the O^6-Cm bond, thus encouraging nucleophilic attack by A63:N1. This predicted resonance is shown in Figure S5 compared to that of O^6-(p-aminomethyl)benzylguanine (ab^{6}G), which is expected to stabilize positive charge more effectively.

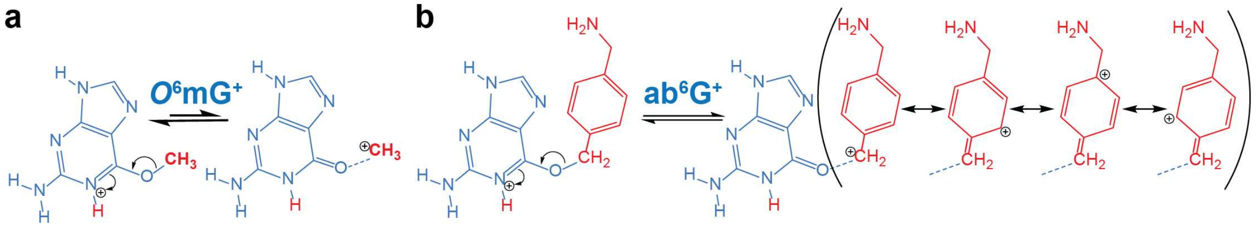

Figure S5. Resonance structure of **(a)** O^6mG$^+$ and **(b)** ab^{6}G$^+$ that promotes O^6-Cm bond cleavage.

The reaction coordinates described above force the system through rare, high-energy states that cannot be observed crystallographically. The rate determining barrier resulting from methyl transfer requires A63:N1 to be sufficiently close to Cm to achieve orbital overlap and initiate nucleophilic attack. To evaluate the requirements for orbital overlap, we computed the average structure of the *ab initio* trajectories representative of the stationary points along the reaction pathway and measured the distances as shown in Figure 3 of the main text with values given in Table S3. The proton transfer (TS1) and methyl transfer (TS2) barriers involve contraction of the distance between C10:N3-O^6mG:N1 and O^6mG:O6-A63:N1 respectively, which subsequently relax in product state similar to what is observed in the crystal structure.

Table S3. Distances (Å) associated with reaction coordinates of key atoms along the reaction pathway.[a]

	Simulation					Expt.
Distance	R_R	R_{TS1}	R_I	R_{TS2}	R_P	R_P
Methyl transfer						
O^6mG:O6-A63:N1	4.56 (0.08) [4.32, 4.80]	4.44 (0.08) [4.22, 4.74]	4.32 (0.07) [4.15, 4.60]	3.94 (0.08) [3.71, 4.17]	4.27 (0.08) [4.03, 4.50]	4.2
O^6mG:O6-Cm	1.42 (0.03) [1.37, 1.54]	1.44 (0.03) [1.37, 1.60]	1.45 (0.03) [1.37, 1.56]	1.84 (0.05) [1.73, 2.00]	2.87 (0.06) [2.75, 3.04]	2.8
A63:N1-Cm	3.14 (0.06) [2.99, 3.32]	3.01 (0.06) [2.86, 3.20]	2.88 (0.05) [2.76, 3.07]	2.09 (0.05) [1.94, 2.27]	1.47 (0.03) [1.41, 1.57]	1.5
Proton transfer						
C10:N3-O^6mG:N1	2.95 (0.05) [2.82, 3.12]	2.67 (0.05) [2.53, 2.85]	2.89 (0.06) [2.73, 3.08]	2.92 (0.05) [2.79, 3.09]	2.93 (0.05) [2.76, 3.09]	2.7
C10:N3-H	1.04 (0.02) [0.98, 1.12]	1.31 (0.04) [1.22, 1.43]	1.84 (0.05) [1.71, 1.98]	1.89 (0.05) [1.77, 2.05]	1.90 (0.05) [1.79, 2.02]	-
O^6mG:N1-H	1.91 (0.05) [1.80, 2.06]	1.36 (0.05) [1.23, 1.53]	1.05 (0.03) [0.95, 1.13]	1.04 (0.02) [0.98, 1.11]	1.03 (0.02) [0.97, 1.14]	-

[a] Values of average distances (R) in Å with (standard deviations) and [minimum, maximum] values calculated from 20 ps of sampling of the converged *ab initio* string (Simulation). Values are compared to experimental distances from the crystal structure of the product (Expt).

Extended data and error analysis for QM/MM and AFE simulations

To construct the activity-pH profile we first computed k_{int} from transition state theory (see Equation. S1) using the free energy barrier derived from QM/MM simulations.The C10U containing system likely reacts at a rate undetectable by experiment as shown in Table 1 of the main text, and has generally higher errors associated with its free energy profile compared to the wild-type (denoted WT). With this in mind, exhaustive QM/MM sampling of the converged string and AFE simulations were reserved for the wild-type system.

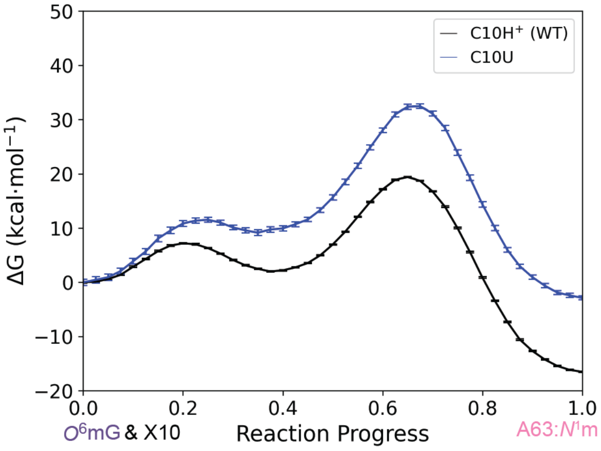

Figure S6. Error analysis of free energy profiles for wild-type (black) and C10U mutant (blue) systems. Error analysis was performed at 200 points along the interpolated curve generated by the ndfes program; however, 40 points are presented here for clarity. X is used to indicate C10H$^+$ or C10U as the reactant state.

To aid in the interpretation of the experimental activity-pH profile, we sought to identify functionally important nucleotides responsible for the derived "apparent pK_a" values in combination with the rate derived from QM/MM simulations. The following alchemical transformations were performed according to the Detailed Computational Methods section and analyzed using the MBAR method to obtain a pK_a shift from the solution value for the general acid (a) as well as nucleophile (b) in the wild-type system. Transformations in which C10 and A63 were alchemically deprotonated were performed in the presence of the O^6mG ligand, and the transformations that were used to construct the activity-pH profile in Figure 4 of the main text are shown in boxes. Each pK_a is derived from two legs corresponding to a transformation of the C10:N3 or A63:N1 hydrogen to a dummy atom in the MTR1 system and in the nucleobase in solution (soln). Knowing the experimental reference pK_a of the species, a pK_a shift is computed by taking the difference in free energies of transformation in solution and MTR1 and converting to pK_a units according to equations (5) and (6).

$$\Delta pK_a^{calc} = -RT \ln \Delta\Delta G = pK_a^{calc}[MTR1] - pK_a^{calc}[soln] \quad \text{(S5)}$$

$$pK_a[MTR1] = pK_a^{expt}[soln] + \Delta pK_a^{calc} \quad \text{(S6)}$$

Using these equations, the higher pK_a of 6.2 was best replicated by deprotonating C10:N3 in the ligand bound form. The pK_a obtained from the deprotonation of A63:N1 while the ligand was bound did not match experiment and was ruled out as the source of the lower pK_a of 5.0.

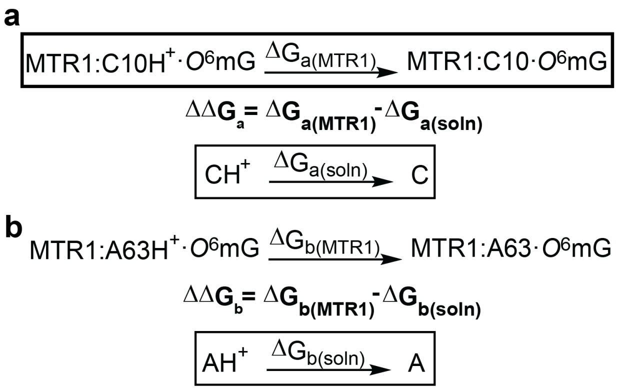

Figure S7. Alchemical transformations that yield pK_a shifts for **(a)** C10 and **(b)** A63 in the ligand bound form of MTR1 and in solution. Transformations that were used to interpret the experimental activity-pH profile are shown in boxes.

The change in charge from 1+ to neutral during the aforementioned alchemical transformations is compensated for by shifting of the hydrogen bonding pattern between O^6mG and C10, which induces rotation of the ligand methyl group. Such rotation is able to be modeled using the enhanced sampling method ACES. To judge the quality of AFE simulations, $\frac{dU}{d\lambda}$ profiles, phase space overlap, and replica exchange acceptance ratios were analyzed. The cubic spline interpolated curve of $\frac{dU}{d\lambda}$ is integrated to determine the free energy of transformation, and should thus be smooth as is true in Figure S8a. Figure S8b demonstrates that the chosen λ schedule ensures adequate phase space overlap that induces a high degree of replica exchange acceptance, thus promoting enhanced sampling.

The quality of enhanced sampling is further reflected by the ability of a replica to complete "round trips." This means a replica has exchanged with all upstream and downstream replicas and returned back to its original value, thus promoting conformational sampling from both sides of a free energy barrier, such as the rotation of the methyl group. Round trip analysis over 5 trials presented in Table S4 demonstrates that the MTR1 systems and the 3-mer references (soln) experienced exhaustive enhanced sampling.

Table S4. Round trip analysis of AFE simulations with replica exchange averaged over 5 trials.[a]

			MTR1			soln		
Nucleotide	Replicas	Exchange attempts	Round trips	Trips /replica	Single trip steps	Round trips	Trips /replica	Single trip steps
C10	25	250000	122.8	4.9	17286.5	1799.6	71.1	1745.6
A63	25	250000	94.7	3.8	27385.4	1776.9	71.1	1743.0

[a]A round trip is achieved when a replica, or λ state, traverses the λ space by replica exchange and returns back to its initial value, thus demonstrating enhanced sampling. Data is reported as the average of 5 independent trials.

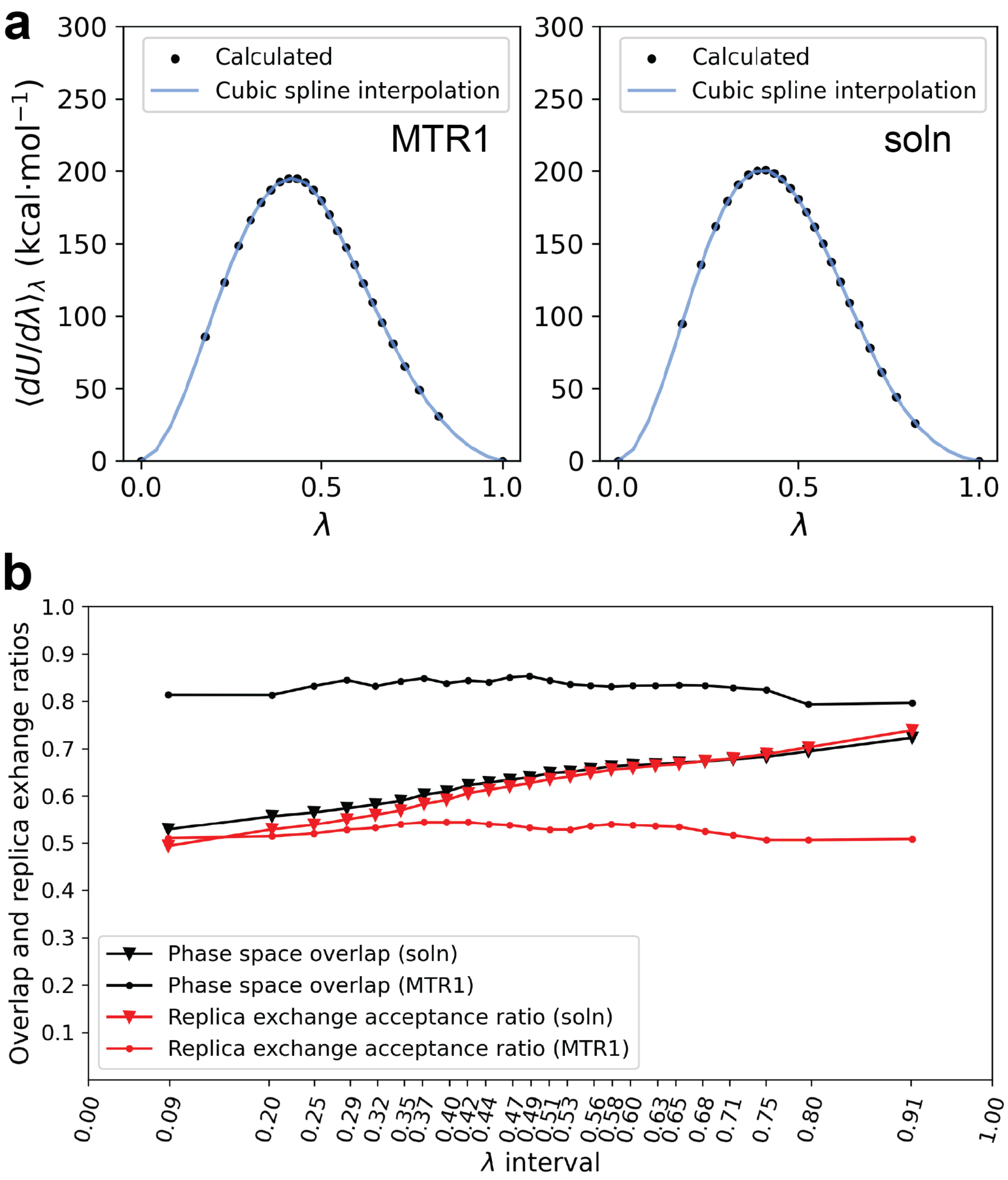

Figure S8. Analysis of the alchemical simulation of C10 protonation in MTR1 and solution (soln) systems for pK_a shift prediction. **(a)** Average $\frac{dU}{d\lambda}$ profile of MTR1 system (left) and solution system (right) over 5 trials with cubic spline projection (blue) that is integrated to determine free energy of transformation. **(b)** Average phase space overlap (black) and replica exchange acceptance ratios (red) in MTR1 (circle) and in solution (triangle) over 5 trials.

REFERENCES

1. Case, D. A., Aktulga, H. M., Belfon, K., Ben-Shalom, I. Y., Berryman, J., Brozell, S. R., Cerutti, D. S., Cheatham III, T. E., Cruzeiro, V. W. D., Darden, T. A. et al. AMBER22 University of California, San Francisco San Francisco, CA (2022).

2. Zgarbová, M., Otyepka, M., Šponer, J., Mládek, A., Banáš, P., Cheatham III, T. E. and Jurečka, P. (2011) Refinement of the Cornell et al. nucleic acids force field based on reference quantum chemical calculations of glycosidic torsion profiles. *J. Chem. Theory Comput.,* **7**, 2886–2902.

3. Pérez, A., Marchán, I., Svozil, D., Sponer, J., Cheatham III, T. E., Laughton, C. A. and Orozco, M. (2007) Refinement of the AMBER force field for nucleic acids: Improving the description of α/γ conformers. *Biophys. J.,* **92**, 3817–3829.

4. Cheatham, III, T. E., Cieplak, P. and Kollman, P. A. (1999) A Modified Version of the Cornell et al. Force Field with Improved Sugar Pucker Phases and Helical Repeat. *J. Biomol. Struct. Dyn.,* **16**(4), 845–862.

5. Wang, J., Wang, W., Kollman, P. A. and Case, D. A. (2006) Automatic atom type and bond type perception in molecular mechanical calculations. *J. Mol. Graph. Model.,* **25**, 247–260.

6. Jakalian, A., Bush, B. L., Jack, D. B. and Bayly, C. I. (2000) Fast, efficient generation of high-quality atomic charges. AM1-BCC model: I. method. *J. Comput. Chem.,* **21**, 132–146.

7. Wang, J., Wolf, R. M., Caldwell, J. W., Kollman, P. A. and Case, D. A. (2004) Development and testing of a general amber force field. *J. Comput. Chem.,* **25**, 1157–1174.

8. Deng, J., Wilson, T. J., Wang, J., Peng, X., Li, M., Lin, X., Liao, W., Lilley, D. M. J. and Huang, L. (2022) Structure and mechanism of a methyltransferase ribozyme. *Nat. Chem. Biol.,* **18**, 556–564.

9. Horn, H. W., Swope, W. C., Pitera, J. W., Madura, J. D., Dick, T. J., Hura, G. L. and Head-Gordon, T. (2004) Development of an improved four-site water model for biomolecular simulations: TIP4P-Ew. *J. Chem. Phys.,* **120**(20), 9665–9678.

10. Joung, I. S. and Cheatham III, T. E. (2008) Determination of alkali and halide monovalent ion parameters for use in explicitly solvated biomolecular simulations. *J. Phys. Chem. B,* **112**, 9020–9041.

11. Darden, T., York, D. and Pedersen, L. (1993) Particle mesh Ewald: An N log(N) method for Ewald sums in large systems. *J. Chem. Phys.,* **98**, 10089–10092.

12. Essmann, U., Perera, L., Berkowitz, M. L., Darden, T., Lee, H. and Pedersen, L. G. (1995) A smooth particle mesh Ewald method. *J. Chem. Phys.,* **103**(19), 8577–8593.

13. Ryckaert, J. P., Ciccotti, G. and Berendsen, H. J. C. (1977) Numerical Integration of the Cartesian Equations of Motion of a System with Constraints: Molecular Dynamics of n-Alkanes. *J. Comput. Phys.,* **23**, 327–341.

14. Berendsen, H. J. C., Postma, J. P. M., van Gunsteren, W. F., Dinola, A. and Haak, J. R. (1984) Molecular dynamics with coupling to an external bath. *J. Chem. Phys.,* **81**, 3684–3690.

15. Warshel, A. and Levitt, M. (1976) Theoretical studies of enzymic reactions: Dielectric, electrostatic and steric stabilization of the carbonium ion in the reaction of lysozyme. *J. Mol. Biol.,* **103**, 227–249.

16. Singh, U. C. and Kollman, P. A. (1986) A combined *ab initio* quantum mechanical and molecular mechanical method for carrying out simulations on complex molecular systems: Applications to the CH_3Cl+Cl^- exchange reaction and gas phase protonation of polyethers. *J. Comput. Chem.,* **7**, 718–730.

17. Nam, K., Gao, J. and York, D. M. (2005) An efficient linear-scaling Ewald method for long-range electrostatic interactions in combined QM/MM calculations. *J. Chem. Theory Comput.,* **1**(1), 2–13.

18. Shirts, M. R. and Chodera, J. D. (2008) Statistically optimal analysis of samples from multiple equilibrium states. *J. Chem. Phys.,* **129**, 124105.

19. Li, P., Jia, X., Pan, X., Shao, Y. and Mei, Y. (2018) Accelerated Computation of Free Energy Profile at ab Initio Quantum Mechanical/Molecular Mechanics Accuracy via a Semi-Empirical Reference Potential. I. Weighted Thermodynamics Perturbation. *J. Chem. Theory Comput.,* **14**(11), 5583–5596.

20. Giese, T. J., Ekesan, Ş. and York, D. M. (2021) Extension of the Variational Free Energy Profile and Multistate Bennett Acceptance Ratio Methods for High-Dimensional Potential of Mean Force Profile Analysis. *J. Phys. Chem. A,* **125**, 4216–4232.

21. Giese, T. J. and York, D. M. (2016) Ambient-Potential Composite Ewald Method for *ab Initio* Quantum Mechanical/Molecular Mechanical Molecular Dynamics Simulation. *J. Chem. Theory Comput.,* **12**, 2611–2632.

22. Chandler, D. (1986) Roles of classical dynamics and quantum dynamics on activated processes occurring in liquids. *J Stat Phys,* **42**, 49–67.

23. Berne, B. J., Borkovec, M. and Straub, J. E. (1988) Classical and modern methods in reaction rate theory. *J. Phys. Chem.,* **92**(13), 3711–3725.

24. Chandler, D. (1978) Statistical mechanics of isomerization dynamics in liquids and the transition state approximation. *J. Chem. Phys.,* **68**, 2959–2970.

25. Doron, D., Kohen, A. and Major, D. T. (2012) Collective Reaction Coordinate for Hybrid Quantum and Molecular Mechanics Simulations: A Case Study of the Hydride Transfer in Dihydrofolate Reductase. *J. Chem. Theory Comput.,* **8**, 2484–96.

26. Henriksen, N. and Hansen, F. Dynamic Solvent Effects: Kramers Theory and Beyond pp. 304–334 Oxford University Press (2018).

27. Lee, T.-S., Tsai, H.-C., Ganguly, A., Giese, T. J. and York, D. M. Robust, Efficient and Automated Methods for Accurate Prediction of Protein-Ligand Binding Affinities in AMBER Drug Discovery Boost Vol. 1397 of ACS Symposium Series, pp. 161–204 (2021).

28. Ganguly, A., Tsai, H.-C., Fernández-Pendás, M., Lee, T.-S., Giese, T. J. and York, D. M. (2022) AMBER Drug Discovery Boost Tools: Automated Workflow for Production Free-Energy Simulation Setup and Analysis (ProFESSA). *J. Chem. Inf. Model.,* **62**, 6069–6083.

29. Tsai, H.-C., Lee, T.-S., Ganguly, A., Giese, T. J., Ebert, M. C., Labute, P., Merz Jr, K. M. and York, D. M. (2023) AMBER Free Energy Tools: A New Framework for the Design of Optimized Alchemical Transformation Pathways. *J. Chem. Theory Comput.,* **19**, 640–658.

30. Lee, T.-S., Tsai, H.-C., Ganguly, A. and York, D. M. (2023) ACES: Optimized Alchemically Enhanced Sampling. *J. Chem. Theory Comput.,* **19**, 472–487.

31. Lee, T.-S., Lin, Z., Allen, B. K., Lin, C., Radak, B. K., Tao, Y., Tsai, H.-C., Sherman, W. and York, D. M. (2020) Improved Alchemical Free Energy Calculations with Optimized Smoothstep Softcore Potentials. *J. Chem. Theory Comput.,* **16**, 5512–5525.

32. Hritz, J. and Oostenbrink, C. (2008) Hamiltonian replica exchange molecular dynamics using soft-core interactions. *J. Chem. Phys.,* **128**, 144121.

33. Yang, M., Huang, J. and MacKerell, Jr., A. D. (2015) Enhanced Conformational Sampling Using Replica Exchange with Concurrent Solute Scaling and Hamiltonian Biasing Realized in One Dimension. *J. Chem. Theory Comput.,* **11**, 2855–2867.

34. Jiang, W. and Roux, B. (2010) Free energy perturbation Hamiltonian replica-exchange molecular dynamics (FEP/H-REMD) for absolute ligand binding free energy calculations. *J. Chem. Theory Comput.,* **6**, 2559–2565.

35. Arrar, M., de Oliveira, C. A. F., Fajer, M., Sinko, W. and McCammon, J. A. (2013) w-REXAMD: A Hamiltonian replica exchange approach to improve free energy calculations for systems with kinetically trapped conformations. *J. Chem. Theory Comput.,* **9**, 18–23.

36. Ekesan, Ş. and York, D. M. (2019) Dynamical ensemble of the active state and transition state mimic for the RNA-cleaving 8-17 DNAzyme in solution. *Nucleic Acids Res.,* **47**(19), 10282–10295.

37. Ganguly, A., Weissman, B. P., Giese, T. J., Li, N.-S., Hoshika, S., Rao, S., Benner, S. A., Piccirilli, J. A. and York, D. M. (2020) Confluence of theory and experiment reveals the catalytic mechanism of the Varkud satellite ribozyme. *Nat. Chem.,* **12**, 193–201.

38. Scheitl, C. P. M., Ghaem Maghami, M., Lenz, A.-K. and Höbartner, C. (2020) Site-specific RNA methylation by a methyltransferase ribozyme. *Nature,* **587**, 663–667.
